# Global scale transcriptome analysis reveals differentially expressed genes involve in early somatic embryogenesis in *Dimocarpus longan* Lour

**DOI:** 10.1186/s12864-019-6393-7

**Published:** 2020-01-02

**Authors:** Yukun Chen, Xiaoping Xu, Zhuanxia Liu, Zihao Zhang, Xu XuHan, Yuling Lin, Zhongxion Lai

**Affiliations:** 1 Institute of Horticultural Biotechnology, Fujian Agriculture and Forestry University, Fuzhou, 350002 China; 2 Institut de la Recherche Interdisciplinaire de Toulouse, IRIT-ARI, 31300 Toulouse, France

**Keywords:** *Dimocarpus longan*, Somatic embryogenesis, Illumina HiSeq, Auxin and cytokinin, Molecular marker gene, qRT-PCR

## Abstract

**Background:**

Somatic embryogenesis (SE) is a process of somatic cells that dedifferentiate to totipotent embryonic stem cells and generate embryos in vitro. Longan SE has been established and wildly used as model system for studying embryogenesis in woody plants, SE-related genes had been characterized. In spite of that, a comprehensive overview of SE at a molecular level is still absent. To understand the molecular mechanisms during longan SE, we examined the transcriptome changes by using Illumina HiSeq from the four distinct developmental stages, including non-embryogenic callus (NEC), embryogenic callus (EC), incomplete compact pro-embryogenic cultures (ICpEC), globular embryos (GE).

**Results:**

RNA-seq of the four samples generated a total of 243.78 million high quality reads, approximately 81.5% of the data were mapped to longan genome. The cDNA libraries of NEC, EC, ICpEC and GE, generated 22,743, 19,745, 21,144, 21,102 expressed transcripts, 1935, 1710, 1816, 1732 novel transcripts, 2645, 366, 505, 588 unique genes, respectively. Comparative transcriptome analysis showed that a total of 10,642, 4180, 5846 and 1785 genes were differentially expressed in the pairwise comparisons of NEC_*vs*_EC, EC_*vs*_ICpEC, EC_*vs*_GE, ICpEC_*vs*_GE, respectively. Among them, plant hormones signalling related genes were significantly enriched, especially the auxin and cytokinin signalling components. The transcripts of flavonoid biosynthesis related genes were mainly expressed in NEC, while fatty acid biosynthesis related genes mainly accumulated in early SE. In addition, the extracelluar protein encoding genes *LTP*, *CHI*, *GLP*, *AGP*, *EP1* were related to longan SE. Combined with the FPKM value of longan nine tissues transcription, 27 SE specific or preferential genes (*LEC1*, *LEC1-like*, *PDF1.3*, *GH3.6*, *AGL80*, *PIN1*, *BBM*, *WOX9*, *WOX2*, *ABI3*, et al.) and 28 NEC preferential genes (*LEA5*, *CNOT3*, *DC2.15*, *PR1–1*, *NsLTP2*, *DIR1*, *PIP1*, *PIP2.1*, *TIP2–1*, *POD-P7* and *POD5* et al.) were characterized as molecular markers for longan early SE. qRT-PCR validation of SE-related genes showed a high correlation between RNA-seq and qRT-PCR data.

**Conclusion:**

This study provides new insights into the role of the transcriptome during early SE in longan. Differentially expressed genes reveal that plant hormones signalling, flavonoid and fatty acid biosynthesis, and extracelluar protein related genes were involved in longan early SE. It could serve as a valuable platform resource for further functional studies addressing embryogenesis in woody plants.

## Background

Longan (*Dimocarpus longan* Lour.), a tropical/subtropical evergreen fruit tree within the Sapindaceae family, native to South China and Southeast Asia, is now widely cultivated in Southeast Asia, South Asia, Australia and Hawaii [1]. Logan embryo development status was close association with the seed size, fruit-set rate, fruit production and quality. Base on the observation of histological and cytological, the change of endogenous hormones and polyamines, proteomics analysis of the isozymes and proteins, molecular biology researches on SE-related genes mRNA differential display, homologous cloning, and expression pattern by qRT-PCR have been used to illuminate the potential regulation mechanism of longan SE [2]. However, elucidating the embryo development mechanism at a molecular level remains a great challenge due to its highly genetic heterozygosity and difficulties in accessibility of early embryos in vivo [3]. Plant SE shares close similarities at almost all development stages to normal zygotic embryogenesis [4, 5], SE has been wildly used as a model system to study the molecular regulation mechanism of early embryogenesis in plants [6]. The longan SE system has been established and extensively used as a model system for investigating embryogenesis in woody plants, which revealed that the concentration of 2,4-D was the key factor in controlling longan high-consistency SE [1, 7, 8].

Over the last few years, the expression profiles of SE related genes and other differentially expressed genes during SE had been extensively excavated by RNA-seq sequencing in various species, including *Gossypium hirsutum* [9–12], *Arabidopsis* [13, 14], Maize [15], Norway spruce [16, 17], Coconut plam [18], Brazilian pine [19], *Eleutherococcus senticosusk* [20], Camphor tree [21], Strawberry [22], Rice [23], *Lilium pumilum* [24], Mangosteen [25], Papaya [26], and *Triticum aestivum* [27]. Meanwhile, the comparative proteome analysis during SE also characterized numerous proteins that associated with SE in many plant species, such as Maize [28], Papaya [29], Cacao [30], Sugarcane [31], *Musa*. spp. [32], and *Gossypium hirsutum* [33]. The transcriptome and proteome analysis of plant SE revealed several molecular regulation mechanisms of SE, and a large number of potential key factors of embryogenesis. Numerous genes and proteins that playing an important role in somatic embryogenesis have been reported, such as *Somatic embryogenesis receptor-like kinase* (*SERK*) [34–36], *Leafy Cotyledon* [36–38], *BABYBOOM* [36, 39, 40], *WUSCHEL* [41, 42], *WUSCHEL homeobox 2* [36, 43], *AGAMOUS-like 15* [44, 45], and *late embryogenesis abundant* (*LEA*) *protein* [26].

To date, the transcript profiling of longan embryogenic callus (EC) had been illuminated by Lai and Lin [46], which revealed numerous embryogenesis-related and reproductive growth related unigenes in EC. Lin and Lai [47] had identified and profiled the conserved and novel miRNA during longan SE by using Solexa sequencing combined with computational, and qRT-PCR methods, and the potential roles of 20 conserved and 4 novel miRNA in longan SE were described by their tissue or stage-specific expression profiling. Recently, longan draft genome sequences become available [48], which provided the comprehensive genomic information for studying the molecular regulation of SE. Transition from NEC to EC, and from EC to somatic embryo are the key steps of SE. However, the molecular regulation mechanisms during longan SE remain largely unknown. To elucidate the molecular mechanism in the transition from NEC to EC, and during early SE by investigating the expression profiling using Illumina RNA-seq technology, and to identify the molecular marker genes during SE. This RNA-seq of comparative transcriptome analysis will gain new insight into the molecular and developmental mechanisms of longan SE.

## Results

### RNA-Seq analysis of longan early SE aligned with the *Dimocarpus longan* draft genome

**Fig. 1:**
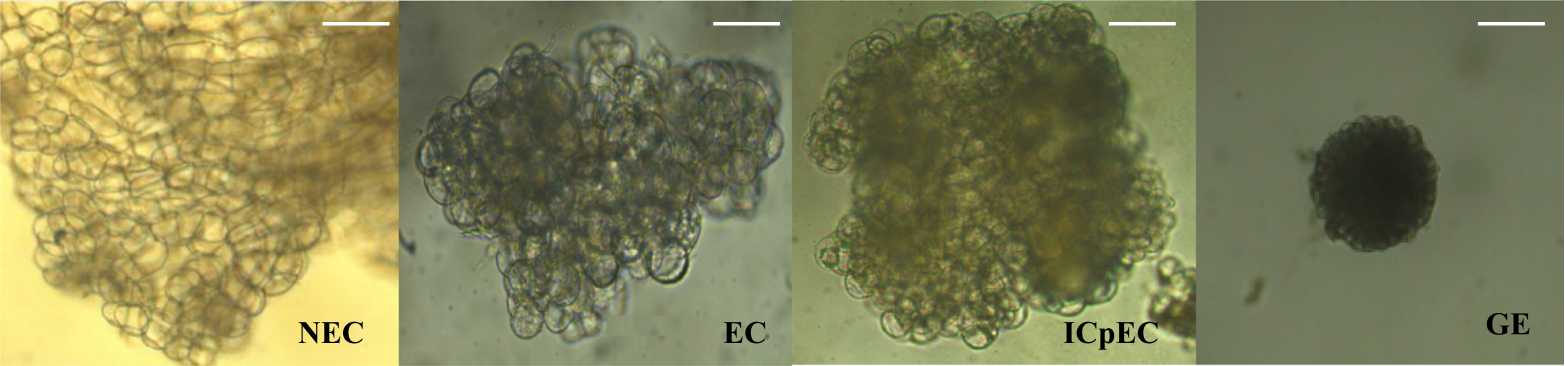
The synchronized cultures during longan SE. NEC: non-embryogenic callus; EC: Friable-embryogenic callus; ICpEC: Incomplete compact pro-embryogenic cultures; GE: Globular embryos. Bars = 50 μm

To provide a comprehensive understanding of longan SE at a transcriptional level, we sequenced the four cDNA libraries constructed from the four in vitro embryo developmental stages (NEC, EC, ICpEC, and GE, Fig. 1). A total of 243,783,126 clean reads (comprising approximately 24.38 G of nucleotides) were obtained after data cleaning and quality checks. After aligned with longan reference genome [48], 48,798,229 (81.62%), 52,623,741 (81.1%), 48,346,067 (81.14%), and 48,871,200 (82.08%) reads in four cDNA libraries were mapped to longan reference genome, respectively. Among these, 44,655,772 (74.69%), 48,333,703 (74.50%), 44,490,292 (74.67%), and 44,924,511 (74.45%) reads were uniquely mapped to one location, respectively. Meanwhile, 34,380,246 (57.51%), 35,386,494 (54.54%), 30,535,088 (51.25%), and 29,214,788 (49.07%) reads in four cDNA libraries were mapped to gene, respectively. A summary of mapping statistics obtained for each sample is given in Table 1.

**Table 1:** Statistics of reads generated by transcriptome sequencing of longan SE

Sample Name	Total Clean reads	Total Reads Map to Genome	Genome Mapping Rate (%)	Total Reads Map to Gene	Gene Mapping Rate (%)	Expressed Transcripts	Expressed Exon	Novel Transcripts	Extend Gene	Alternative Splicing
NEC	59,785,854	48,798,229	81.62	34,380,246	57.51	22,743	110,864	1935	10,281	35,084
EC	64,876,258	52,623,741	81.11	35,386,494	54.54	19,745	103,200	1710	9092	19,056
ICpEC	59,580,846	48,346,067	81.14	30,535,088	51.25	21,144	107,592	1816	9197	36,446
GE	59,540,168	48,871,200	82.08	29,214,788	49.07	21,102	107,971	1732	9638	39,768

The transcribed regions/units of four different stages samples were constructed independently, generated 22,743, 19,745, 21,144, and 21,102 expressed transcripts, showed 57.89, 50.26, 53.82, and 53.71% overlapped with longan genome (39,282 genes), respectively. After filtering out short sequences which less than 180 bp and low sequencing depth that lower than two, 1935, 1710, 1816, and 1732 novel transcripts in four samples were detected, respectively. Among these, 1025, 819, 832, and 806 novel genes were identified as coding RNAs, and 910, 891, 984, and 926 novel genes were identified as non-coding RNAs in longan genome.

**Fig. 2:**
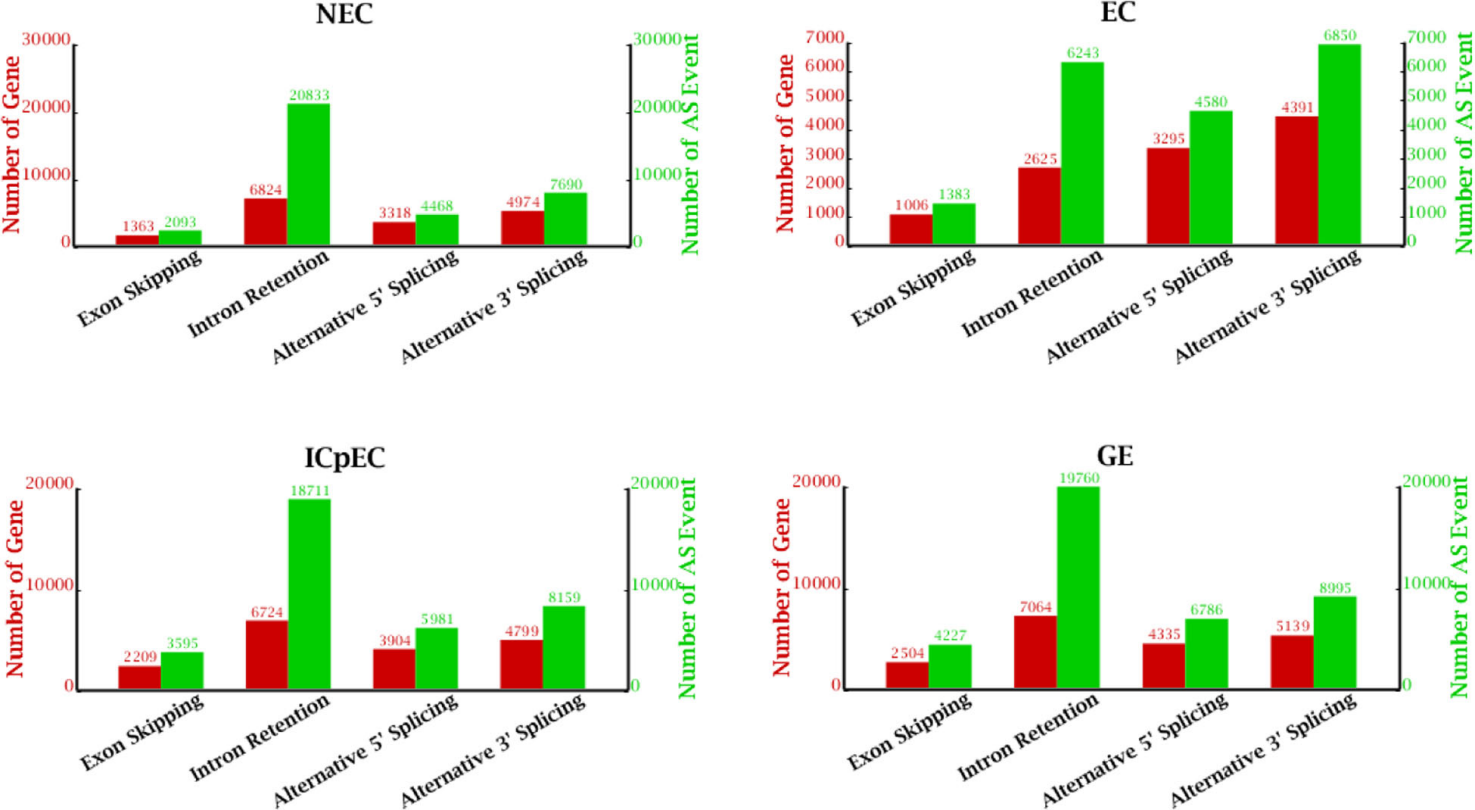
Alternative splicing events in the four stages of SE

Alternative splicing (AS) events represented in our transcriptome were predicted by TopHat2. We analyzed the exon level of the four samples, 110,864, 103,200, 107,592, and 107,971 expressed exon were detected (Table 1). A total of 130,354 AS events were checked across the four stages, including exon skipping, intron retention, alternative 5′ splicing and alternative 3′ splicing. The largest number of AS events were detected in GE (39,768), followed by ICpEC (36,446), and NEC (35,084), and the smallest in EC (19,056). Exon skipping is the least type in all samples, and intron retention is the most popular type of AS events in NEC, ICpEC and GE (Fig. 2).

### Global analysis of gene expression across the four distinct developmental processes

**Fig. 3:**
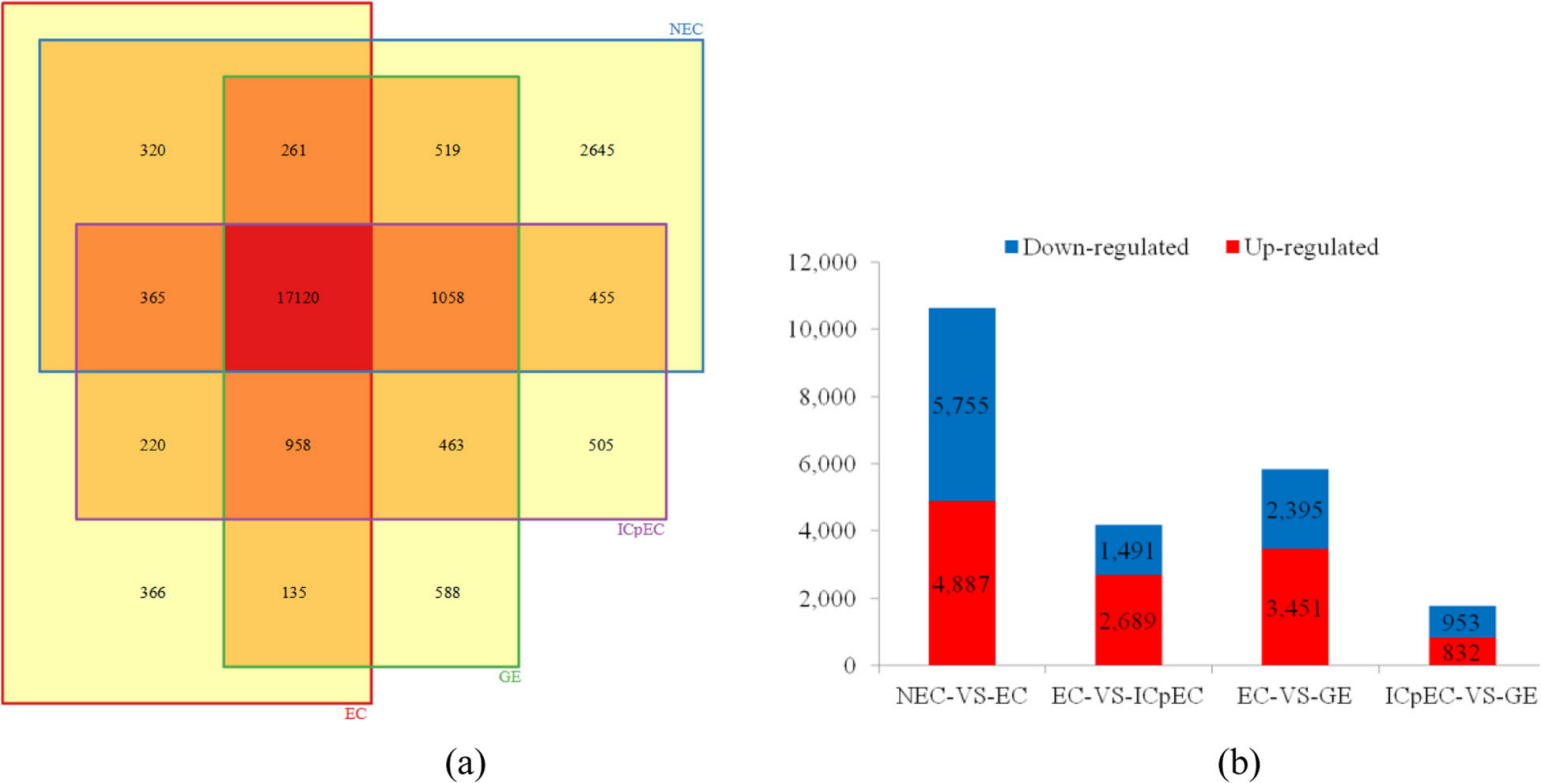
Statistical analysis of differentially expressed unigenes in NEC and early SE stages. **a** The *venn diagram* of expressed genes in four developmental stages. **b** Statistic of Up/Down regulated genes in pairwise comparisons of NEC_*vs*_EC, EC_*vs*_ICpEC, EC_*vs*_GE, and ICpEC_*vs*_GE

There were 22,743, 19,745, 21,144, and 21,102 expressed genes in NEC, EC, ICpEC, and GE stage. Among these, more than 75.3% of the expressed genes were present in all four developmental stages, while 2645 genes were only expressed in NEC. However, only 366, 505 and 588 genes were unique present in EC, ICpEC, and GE stage, respectively (Fig. 3a), which suggested that distinct spatial transcriptional patterns were present in the four developmental processes. To evaluate the differences of molecular response among four samples, gene expression were normalized to FPKM by RSEM software. After filtering with FPKM> 60, a total of 2961 (11.40%), 3445 (13.26%), 3445 (13.26%), and 3442 (13.25%) genes were highly expressed in NEC, EC, ICpEC, and GE, respectively (Table 2). The Top10 most enriched (FPKM) genes were range from 5476 to 58,812, 2766 to 15,114, 2343 to 10,330, and 2091 to 4004, respectively. The top 20 most expressed genes from the four libraries were shown in Tables 3, SE-related genes such as *leafy cotyledon 1* (*LEC1*), *leafy cotyledon 1-like* (*L1L*), *Protodermal factor 1 (PDF1)*, *lipid transfer protein (LTP)*, *Heat-Shock protein 90 (HSP90)*, *chitinase* (*CHI*), *Indole-3-acetic acid-amido synthetase GH3.6*, *glutathione S-transferase* (*GST*), *root meristem growth factor 3* (*RGF3*) were highly expressed in EC, ICpEC or GE stage.

**Table 2:** Gene expression levels given in FPKM during longan SE

FPKM Interval	NEC	EC	ICpEC	GE
≤0.1	3900(15.01%)	6910(26.60%)	5364(20.65%)	5391(20.75%)
0.11–1	3587(13.81%)	3075(11.84%)	3241(12.48%)	3384(13.03%)
1.01–3	2706(10.42%)	1957(7.53%)	2439(9.39%)	2362(9.09%)
3.01–15	6440(24.79%)	4774(18.38%)	5413(20.84%)	5208(20.05%)
15.01–60	6384(24.57%)	5817(22.39%)	6076(23.39%)	6191(23.83%)
60.01–100	1278(4.92%)	1431(5.51%)	1601(6.16%)	1573(6.06%)
≥100	1683(6.48%)	2014(7.75%)	1844(7.10%)	1869(7.19%)

**Table 3:** The top 20 most expressed genes in NEC, EC, ICpEC, GE library

NO.	Gene_id	FPKM_NEC	Description
1	Dlo_008315.1	58,812.43	repetitive proline-rich cell wall protein 2
2	Dlo_019949.1	36,215.45	Late embryogenesis abundant protein Lea5
3	Dlo_008311.1	11,187.75	unknow protein
4	Dlo_028175.1	10,885	unknow protein
5	Dlo_011615.1	10,317.69	extensin-2-like
6	Dlo_030517.1	8931.79	chitinase CHI
7	Dlo_004649.1	7800.33	metallothionein
8	Dlo_024177.1	6055.72	chitinase
9	Dlo_017033.1	5645.16	pathogenesis-related protein 1
10	Dlo_008997.3	5476.22	unknow protein
11	Dlo_009172.1	5469.19	osmotin-like protein I
12	Dlo_021620.1	4483.38	peroxidase 4
13	Dlo_003142.1	4116.17	unknow protein
14	Dlo_030075.1	3732.29	Wound-induced protein WIN1 precursor
15	Dlo_030519.1	3587.55	chitinase CHI
16	Dlo_022694.1	3546.1	14 KDa proline-rich protein DC2.15-like
17	Dlo_030516.1	3288.7	chitinase
18	Dlo_030074.1	3170.89	PR-4 protein
19	Dlo_011076.1	2572.89	ubiquitin C
20	Dlo_011004.1	2367.71	non-specific lipid-transfer protein 2-like
NO.	Gene_id	FPKM_EC	Description
1	Dlo_030812.1	15,114.88	Protodermal factor 1.3 PDF1.3
2	Dlo_013012.1	7392.37	lipid transfer protein
3	Dlo_030517.1	5117.6	chitinase CHI
4	Dlo_025725.1	4537.79	Pollen-specific protein C13 precursor
5	Dlo_020986.1	4136.45	Indole-3-acetic acid-amido synthetase GH3.6
6	Dlo_011615.1	4031.23	extensin-2-like
7	Dlo_021620.1	3793.82	peroxidase 4
8	Dlo_032035.1	2962.58	histone H4-like
9	Dlo_031913.1	2959.02	lipid binding protein
10	Dlo_003789.1	2766.68	EXORDIUM-like 2 EXL2
11	Dlo_005176.1	2363.28	omega-6 fatty acid desaturase
12	Dlo_026048.1	2219.4	root meristem growth factor 3 RGF3
13	Dlo_033433.1	2202.74	unknow
14	Dlo_019526.1	2145.11	unknow
15	Dlo_017203.1	2144.67	Hsp90
16	Dlo_026351.1	2131.12	peptidase
17	Dlo_017092.1	2118.54	transcription factor leafy cotyledon1
18	Dlo_020190.1	2035.05	cysteine protease
19	Dlo_007905.1	2000.4	small ubiquitin-related modifier 2-like
20	Dlo_012332.1	1967.95	26S proteasome complex subunit DSS1
NO.	Gene_id	FPKM_ICpEC	Description
1	Dlo_030812.1	10,330.16	protodermal factor 1.3
2	Dlo_026048.1	6620.49	root meristem growth factor 3 RGF3
3	Dlo_031913.1	4694.68	lipid binding protein
4	Dlo_013012.1	4301.99	lipid transfer protein
5	Dlo_008315.1	4140.34	proline-rich cell wall protein 2-like PRP2
6	Dlo_032146.1	3401.87	NADH dehydrogenase (ubiquinone)1 beta subcomplex 7
7	Dlo_028379.1	2815.75	dehydrin 1
8	Dlo_025725.1	2772.74	Pollen-specific protein C13 precursor
9	Dlo_020986.1	2439.09	Indole-3-acetic acid-amido synthetase GH3.6
10	Dlo_021620.1	2343.85	peroxidase 4
11	Dlo_019476.1	2300.16	unknow
12	Dlo_028328.1	2106.48	high mobility group box 1
13	Dlo_017203.1	1958.55	Hsp90
14	Dlo_019638.1	1958.53	elongation factor 1-alpha
15	Dlo_030608.1	1823.94	unknow
16	dlo_034323.1	1818.75	histone H1
17	Dlo_011615.1	1789.67	extensin-2-like
18	Dlo_010406.1	1773.3	transcription factor BTF3
19	Dlo_017539.1	1684.81	histone H2B.1-like
20	Dlo_030517.1	1598.6	chitinase CHI
NO.	Gene_id	FPKM_GE	Description
1	Dlo_013012.1	4004.61	lipid transfer protein
2	Dlo_026048.1	3762.46	root meristem growth factor 3 RGF3
3	Dlo_021620.1	3304.97	peroxidase 4
4	Dlo_031913.1	2957.57	lipid binding protein
5	Dlo_032146.1	2784.3	NADH dehydrogenase (ubiquinone)1 beta subcomplex 7
6	Dlo_028379.1	2676	dehydrin 1
7	Dlo_014867.1	2300.44	argonaute 4
8	Dlo_008315.1	2218.56	proline-rich cell wall protein 2-like PRP2
9	Dlo_030608.1	2100.52	unknow
10	Dlo_012964.1	2091.04	extensin, proline-rich protein
11	Dlo_032870.1	2072.74	glutathione S-transferase parC-like
12	Dlo_025725.1	2018.61	Pollen-specific protein C13 precursor
13	Dlo_019476.1	1997.45	unknow
14	Dlo_015927.1	1907.93	unknow
15	Dlo_030812.1	1899.07	protodermal factor 1.3
16	Dlo_028328.1	1848.42	high mobility group box 1
17	Dlo_018634.1	1743.25	60S ribosomal protein L27Ae
18	Dlo_019638.1	1709.96	elongation factor 1-alpha
19	Dlo_017203.1	1696.73	Hsp90
20	Dlo_020821.1	1665.7	leafy cotyledon1-like

To reveal the potential key genetic factors involved in early SE, we filtered the significantly differentially expressed genes (DEGs) with |log_2_^fold change^| ≥ 1 and FDR < 0.001 between these four pairwise comparisons as follow: NEC_*vs*_EC, EC_*vs*_ICpEC, EC_*vs*_GE, and ICpEC_*vs*_GE. Among these four comparisons (Fig. 3b), a total of 10,642, 4180, 5846 and 1785 DEGs were identified, respectively. Compared with NEC, EC had 4887 up-regulated and 5755 down-regulated genes. Compared with EC, ICpEC had 2689 up-regulated and 1491 down-regulated genes, GE had 3451 up-regulated and 2395 down-regulated genes. Compared with ICpEC, GE had 832 up-regulated and 953 down-regulated genes. DEGs analysis revealed that longan transcriptome undergoes significantly dynamic changes during SE, particularly during the transition period from NEC to EC. Therefore, the longan SE transcriptome datasets given here may serve as a valuable molecular resource for future studies.

### Functional classification of DEGs base on GO and KEGG

To evaluate the potential functions of the DEGs, we used GO terms assignment to classify the functions of DEGs in pairwise comparisons under three GO main categories: biological process, cellular component and molecular function (Additional file 1: Figure S1). In all pairwise comparisons, the term with the largest proportion in “biological process” was ‘metabolic process’, followed by ‘cellular process’, ‘single-organism process’, ‘respond to stimulus’ and ‘localization’, the term with the largest proportion in “cellular component” were ‘cell’ and ‘cell part’, followed by ‘organelle’ and ‘membrane’, the term with the largest proportion in “molecular function” was ‘catalytic activity’, followed by ‘binding’, ‘transporter activity’, ‘molecular transducer activity’ and ‘nucleic acid binding transcription factor activity’ .

To investigate the biological pathways of the DEGs, we used the KEGG database to classify the DEGs function with emphasis on biological pathways (Additional file 2: Figure S2). According to KEGG annotation, 6516 DEGs (NEC_*vs*_EC) were assigned to 128 pathways, 2514 DEGs (EC_*vs*_ICpEC) were assigned to 126 pathways, 3555 DEGs (EC_*vs*_GE) were assigned to 126 pathways, 1062 DEGs (ICpEC_*vs*_GE) were assigned to 111 pathways. The annotated changes in all comparisons were mainly enriched in ‘metabolic pathway’ (21.38, 22.43, 23.12 and 25.52%, respectively), ‘biosynthesis of secondary metabolites’ (11.97, 11.46, 11.70 and 14.52%, respectively), ‘plant-pathogen interaction’ (8.01, 8.23, 7.59 and 6.40%, respectively) and ‘plant hormone signal transduction’ (5.22, 5.41, 5.40 and 8.38%, respectively) pathway. Furthermore, dozens of genes involved in ‘flavonoid biosynthesis’, ‘phenylpropanoid biosynthesis’, ‘zeatin biosynthesis’, ‘fatty acid biosynthesis’ and ‘biosynthesis of unsaturated fatty acids’.

### Differential expression analysis of plant hormones signaling pathway related genes during longan SE

**Fig. 4:**
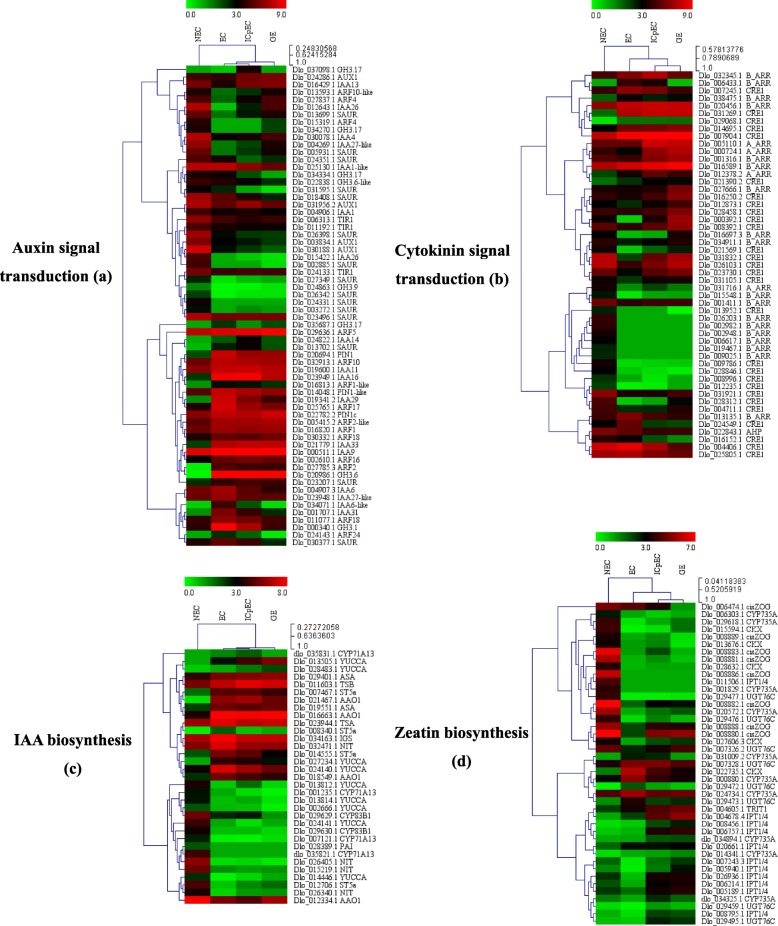
Heatmap of the differentially expressed genes in auxin and cytokinin signaling pathway during longan SE. **a** Auxin signal transduction; **b** Cytokinin signal transduction; **c** IAA biosynthesis; **d** Zeatin biosynthesis. The heatmap was clustered by *pearson* method of Mev4.90 software. Heatmap indicate the gene expression level by Log2[FPKM+ 1] with a rainbow color scale, each row represents a single gene, the IDs and names of selected DEGs are indicated to the right of the histograms, and each column represents a sample

Based on the KEGG and other annotation, plant hormone signal transduction, zeatin biosynthesis and tryptophan metabolism were the representative pathways in our study. A large number of genes invovled in auxin (97 DEGs) and cytokinin (94 DEGs) biosynthesis and signal transduction pathway were differentially expressed when compared EC with NEC (Additional file 3: Figure S3) and early SE. For example, the expression level of *PIN1*, *IAA* (*IAA6*, *IAA6-like*, *IAA9*, *IAA11*, *IAA14*, *IAA16*, *IAA29*, *IAA31* and *IAA33*), *ARFs* (*ARF1*, *ARF1-like*, *ARF2*, *ARF2-like*, *ARF5*, *ARF10*, *ARF16*, *ARF17*, *ARF18*, *ARF18–1* and *ARF24*), *GH3* (*GH3.6*, *GH3.1*, *GH3.17*), and three *SAUR*, genes involved in auxin signal transduction, were significantly up-regulated from NEC to EC, most of them remained highly expression in EC, ICpEC and GE stages. Nevertheless, *AUX1*, *TIR1*, *IAA* (*IAA1*, *IAA4*, *IAA13*, *IAA26*, *IAA26-like*, *IAA27*), *ARFs* (*ARF4*, *ARF4-like*, *ARF10-like*), *GH3.9* and *GH3.17-like*, and 12 *SAUR* were mainly expressed in NEC stage and down-regulated in EC. From EC to ICpEC and GE stages, *AUX1* (Dlo_024286.1, Dlo_031956.2), *IAA* (*IAA4*, *IAA14*, *IAA26-like*, *IAA27*, *IAA13*), *ARFs* (*ARF4*, *ARF4-like*, *ARF10-like*), two *SAUR* showed noteworthy up-regulated expression (Fig. 4a). In IAA biosynthesis, except *PAI*, Trp synthesis key genes *ASA*, *IGS*, *TSA*, *TSB*, were up-regulated in EC and remained high expression during early SE. *CYP83B1*, one *ST5a*, five *YUCCAs*, three *CYP71A13* and *NIT* showed NEC-specific expression pattern. Three *YUCCAs*, three *AAO1*, one *NIT*, *CYP71A13* and three *ST5a* were up-regulated in EC and remained high during early SE, and *YUCCA*_Dlo_013505.1 kept up-regulated during early SE (Fig. 4b).

As showed in Fig. 4c, *TRIT1*, a gene involved in *cis*-zeatin synthesis was up-regulated from NEC to GE. *CisZOG* family involved in *Cis*-zeatin O-glycosylation were highly expressed in NEC, and significantly down-regulated from NEC to EC. During early SE, five *CisZOG* were up-regulated from EC to ICpEC, four *CisZOG* were down-regulated from ICpEC to GE. In *trans*-zeatin biosynthesis, six *IPT1*,*4*, five *CYP735A*, four *CKX*, three *UGT76C* were noteworthy down-regulated from NEC to EC; two *IPT1*,*4*, four *CYP735A*, one *CKX*, three *UGT76C* were up-regulated in EC. During early SE, *IPT1*,*4* family, five *CYP735A*, two *CKX*, four *UGT76C* were up-regulated during early SE with minimal FPKM. Among the cytokinin signal pathway, two *A_ARR*, 10 *B_ARR*, 15 *CRE1* were mainly expressed in NEC, and down-regulated in EC. One *A_ARR*, five *B_ARR*, seven *CRE1* were up-regulated in EC. 13 *CRE1*, seven *B_ARR* and all *A_ARR* showed up-regulated expression during early SE, two *B_ARR* and five *CRE1* were down-regulated during early SE (Fig. 4d).

In addition, numerous genes involved in abscisic acid, gibberellin, ethylene, salicylic acid, jasmonic acid and brassinosteroid signal transduction pathway were differentially expressed during longan SE (Additional file 4: Figure S4; Additional file 5: Table S1 a-h). Such an observation suggested an essential role of hormones and their complicated crosstalk during early SE. Therefore, the plant hormones signaling pathway may be the key regulator during longan early SE.

### Flavonoids and fatty acid biosynthesis related genes were differential expressed during longan SE

**Fig. 5:**
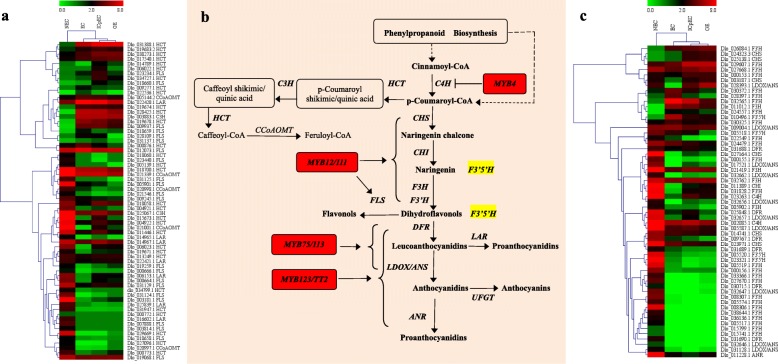
Simplified diagram of flavonoid biosynthetic pathway. **a** Cluster analysis of expression profiles of *HCT*, *C3H*, *CCoAOMT*, *FLS* and *LAR*. **b** Simplified diagram of flavonoid biosynthetic pathway. **c** Cluster analysis of expression profiles of *C4H*, *CHS*, *CHI*, *F3H*, *F3’H*, *F3’5’H*, *DFR*, *LDOX/ANS* and *ANR*. The heatmaps was clustered by pearson method of Mev4.90 software. Heatmaps indicate the gene expression levels by Log2 [FPKM+ 1] with a rainbow color scale, each row represents a single gene, and each column represents a sample. The IDs and names of selected DEGs are indicated to the right of the histograms

Flavonoid biosynthesis and fatty acid biosynthesis were the representative KEGG pathways, a total of 125 significant DEGs were assigned to ‘flavonoid biosynthesis’ across the early SE processes (Fig. 5). In the transition from NEC to EC, the flavonoid biosynthesis key genes, *C4H*, *CHS*, *CHI*, *F3H*, *F3’5’H*, *DFR*, *LDOX/ANS*, *ANR*, *LAR*, *CCoAOMT* were mainly expressed in NEC, while drastic down-regulated from NEC to EC and remained very low expression level in ICpEC and GE stages, except that *F3H_*Dlo_011012*.*1, *F3’5’H_* Dlo_010496.1*, LAR_* Dlo_022420.1, *CCoAOMT_* Dlo_005144.2 were up-regulated in EC, but down-regulated during early SE. Besides, most of the *FLS* and *F3’H* family were mainly expressed in NEC, significantly down-regulated in EC and kept low FPKM during early SE, especially, 15 *F3’H* and 9 *FLS* belonged to NEC-specific genes. Only four *FLS* and six *F3’H* were up-regulated from NEC to EC and then down-regulated or kept low expression level during early SE (Additional file 6: Table S2).

Several *R2R3-MYB* transcription factors are involve in the regulation of flavonoid biosynthesis in *Arabidopsis* [49–51]. For example, *AtMYB11*, − *12*, − *111* regulated flavonol biosynthesis by up-regulated *CHS*, *CHI*, *F3H*, *F3’H* and *FLS* [49, 52]. *AtMYB75*, − *90*, − *113*, − *114* controlled anthocyanin biosynthesis in vegetative [53]. *AtMYB123* controlled the biosynthesis of proanthocyanidins in the seed coat [54]. *MtMYB5*, − *14* played the key role in seed coat polymer biosynthesis [55]. *AtMYB4* negative controlled sinapate ester biosynthesis through down-regulated *C4H* in a UV-dependent manner [56]. In our study, 11 *R2R3-MYB* transcripts were differentially expressed. During longan SE, *MYB12* and *MYB111* were barely expressed in NEC, significant up-regulated from NEC to EC and remained high during early SE. *MYB75*, *MYB113*, *MYB4* and *MYB123* were significant down-regulated in EC, and kept relative low expression during early SE.

The fatty acid composition rapidly changed during SE in *Daucus carota* [57]*,* and *Gossypium hirsutum* [33]. In our study, a total of 35 fatty acid biosynthesis related genes were differently expressed during SE (Additional file 7: Table S3). From NEC to EC, except *ACCase* (Dlo_000360.1), three *FabG*, two *FabZ*, *SAD* (Dlo_031652.1), most of the *ACCase*, *FabD*, *FabF*, *FabG*, *FabZ*, *FabI*, *FatB* and *SAD* were significantly up-regulated in EC. During early SE, most of the DEGs remained high expression, part of them with slightly up/down-regulated expression. For example, *ACCase* (Dlo_023270.1) and *SAD* (Dlo_019646.1) were up-regulated from NEC to EC, and highly expressed during early SE. Our results indicated that flavonoids were mainly expressed in NEC, while fatty acid were mainly accumulated in early SE stages, especially in EC.

### Extracellular protein encoding genes effect on the transition from NEC to EC

It had been reported that extracellular protein germins and germin-like (GLPs), Arabinogalactan proteins (AGPs), chitinases (CHIs), lipid transfer proteins (LTPs) and glycoprotein were critical to SE, and can be served as protein marker during early SE [58]. In our study, 16 *CHIs* were differentially expressed, and most of them were preferential expressed in NEC, and remarkable down-regulated in EC, only seven *CHIs* were up-regulated during early SE with low FPKM. Among the 14 identified *LTPs*, only *LTP* (Dlo_013012.1, Dlo_013014.1) were highly and specific expressed in early SE, most of them were mainly expressed in NEC and down-regulated from NEC to EC. Meanwhile, 12 *GLPs* and two secreted glycoprotein genes (*EP1*-like) were mainly expressed in NEC and kept very low FPKM during early SE. Except *AGP10* was first up-regulated in EC and down-regulated during early SE, most of the *AGPs* were down-regulated in EC, and kept relative low expression level during early SE (Additional file 8: Table S4). The results indicated that most of the extracellular protein encoding genes were mainly expressed in NEC, they were predicted to involve in the transition from NEC to EC.

### Characterization of molecular markers for longan SE

Several genes have been reported to molecular marker of SE, such as *somatic embryogenesis receptor-like kinase* (*SERK*), *leafy cotyledon1* (*LEC1*), *BABYBOOM* (*BBM*), *wuschel* (*WUS*), *WUS-homeobox* (*WOX*). In order to characterize the full-scale of molecular markers for early SE, the comparative analysis of FPKM in nine tissues of longan [48], including root, stem, leaf, flower, flower bud, young fruit, pericarp, pulp and seed [48] were employed to select the molecular marker genes during SE. For our purposes here, it is crucial to identify the reliable molecular marker genes for distinguishing NEC stage from EC, ICpEC and GE stages. In our study, several embryogenesis-labeled genes that had been reported previously were differentially expressed in each stage (Additional file 9: Table S5). However, some of them showed down-regulated or slightly up-regulated in EC, and kept low expression level from NEC to GE, such as *late embryogenesis abundant protein* (*LEA14A*, *LEAD34*, LEA76), *SERK1*, *SERK3*, *WUS*, *WOX5*, *WOX3*, *AIL6*, *AGL15*, *CLV1*, *EMB8*, suggesting that they were unseemly markers for longan SE.

In our study, a total of 55 genes were identified as representative molecular markers, which were closely related to SE, can be classified as two main categories: NEC markers and SE molecular markers by their specific expression profiles in all test-samples (Table 4). The SE marker genes were barely or undetected in NEC, highly expressed during early SE, they also can be divided into SE-specific and SE-expressed genes. The SE-specific genes were highly transcribed only in somatic embryos, including *LEC1*, *LEC2*, *WOX9*, *WOX2*, *Agamous-like 80* (*AGL80*), *PIN-FORMED1* (*PIN1*), *BBM*, *PLETHORA2* (*PLT2*), *mannan endo 1,4-beta-mannosidase7* (*MAN7*), *Glycine-rich protein 5* (*GRP-5*), *GRF*-*interacting factor 2* (*GIF2*), *root meristem growth factor 3* (*RGF3*), *60S ribosomal protein L17e* (*RPL17e*), *zeta-carotene desaturase* (*ZDS*), *3-ketoacyl-CoA synthase* (*KCS*), *CYP78A5*, *CYP87A3* and three unknown genes (*DlU1*, *DlU2*, *DlU3*) (Table 4). These SE-specific genes might play a key role in longan SE. The SE-expressed genes were similar to SE-specific genes, except that these genes also highly expressed in one or some tested tissues included in this study, including *LEC1-like* (*L1L*), *ABA-insensitive protein 3* (*ABI3*), *FUSCA3* (*FUS3*), *Indole-3-acetic acid-amido synthetase* (*GH3.6*), *Protodermal factor 1.3* (*PDF1.3*), *Lipid transfer protein* (*LTP*, Dlo_013012.1) and *Lipid binding protein* (*LBP*). For instance, *L1L*, *FUS3* and *ABI3* showed very strong transcription level not only in somatic embryos but also in seed. *GH3.6* was highly expressed in flower, *PDF1.3* and *LBP* showed high expression level in pulp, *LTP* also highly transcribed in pulp, flower bud, flower and stem, suggesting their multifunctional on SE and other development processes (Table 4).

**Table 4:** The expression profile of candidate markers during SE and in nine tissues of longan

Gene_id	Gene name	FPKM value													Putative protein function/homology
		NEC	EC	ICpEC	GE	Root	Stem	Leaf	Flower	Flower bud	Young fruit	Pericarp	Pulp	Seed	
Dlo_017092.1	*LEC1*	0	2118.54	1262.61	727.62	0	0	0	0	0	0	0	0	1.52	leafy cotyledon 1 transcription factor
Dlo_020821.1	*L1L*	0.21	511.04	393.39	1665.7	0	0	0	0	0	0	0	0	209.01	leafy cotyledon 1-like protein
Dlo_022316.1	*WOX9*	1.34	1602.12	1424.69	665.41	0.3	0	0	0	0.52	1.5	0	5.98	0.67	WUSCHEL-related homeobox 9
Dlo_032045.1	*WOX2*	0.48	141.61	83.91	59.36	0	0	0	0	0	0	0	0	0.17	WUSCHEL-related homeobox 2
Dlo_011527.1	*BBM*	6.28	367.26	484.12	511.04	9.86	0.28	0.1	0.12	0.19	0.34	0.14	0	0.67	AP2/ERF, BABYBOOM
Dlo_004646.1	*PLT2*	3.94	491.37	610.1	759.4	0.41	0	0	0	0	0	0	0	0	AP2/ERF, PLT2
Dlo_012160.1	*ABI3*	0	82.94	177.78	374.51	0	0	0	0.13	13.28	0	0	0	119.83	ABA-insensitive protein 3
Dlo_022372.1	*LEC2*	0	45.35	55.63	51.22	0.11	0	0	0	0	0	0	0	0.4	leafy cotyledon 2 transcription factor
Dlo_024008.1	*FUS3*	0	5.75	37.93	260.7	0	0	0	0	0.35	0	0	0	60.65	FUSCA3
Dlo_017585.1	*AGL80*	0	829.45	590.14	278.12	0.62	0.58	0.82	0.49	0	0.29	0.84	0.58	1.56	Agamous-like MADS-box protein 80
Dlo_020986.1	*GH3.6*	0.06	4136.45	2439.09	1630.99	0	1.2	0.06	843.22	0.32	0	0.25	0.2	0.14	IAA-amido synthetase GH3.6
Dlo_020694.1	*PIN1*	3.23	216.9	96.91	100.29	0	0	0	0.07	0	0	0	0	3.04	Auxin efflux carrier component 1
Dlo_030812.1	*PDF1.3*	15.81	15,114.88	10,330.16	1899.07	0.68	0	0.16	2.4	60.18	76.88	21.19	680.91	16.67	Protodermal factor 1.3
Dlo_027182.2	*GRP-5*	0	1699.47	1094.49	874.66	0	0.35	0	0	0.32	0	0	0	8.03	glycine-rich protein 5
Dlo_032565.1	*CYP78A5*	0.97	320.4	102.08	12.72	31.26	0	1.85	0.09	6.93	1.78	0.4	0.47	4.39	Cytochrome P450 family 78A5
Dlo_017331.1	*CYP87A3*	0	274.33	130.99	25.01	0	0.11	0	5.98	0.63	0.22	0.22	4.42	0.18	Cytochrome P450 family 87A3
Dlo_026048.1	*RGF3*	0	2219.4	6620.49	3762.46	0	0	0	3.27	1.96	0	0	0	0	root meristem growth factor 3
Dlo_026819.1	*GIF2*	0	726.78	454.72	339.9	0	0.94	0	1.08	6.39	0.45	0.3	1.79	12.93	GRF1-interacting factor 2, GIF2
Dlo_013012.1	*LTP*	6.56	7392.37	4301.99	4004.61	3.92	1182.18	5.16	1241.54	1954.47	354.17	62.65	4225.76	67.83	Lipid transfer protein
Dlo_031913.1	*LBP*	3.92	2959.02	4694.68	2957.57	1.19	25.49	12.3	28.54	27.78	32.46	4.93	428.03	5.16	Lipid binding protein
Dlo_025851.1	*MAM7*	3.43	1112.42	436.33	119.05	0	0	0	0	0	0	0	0	0	mannan endo-1,4-beta-mannosidase 7
Dlo_029005.1	*ZDS*	0	1819.85	1213.68	932.22	0.15	0.15	0.11	0.76	0.81	20.69	0.42	14.54	2.2	zeta-carotene desaturase
Dlo_023272.1	*KCS*	0	511.05	371.46	205.05	0	0	0.08	0	0.1	1.36	0	1.49	0.09	3-ketoacyl-CoA synthase (fatty acid)
Dlo_032570.1	*RPL17*	0	1081.06	1123.65	990.78	48.99	40.71	0	0	3.48	3.17	0	0	0	60S ribosomal protein L17
Dlo_033433.1	*DlU1*	3.52	2202.74	1163.64	830.54	14.46	1.61	0	0	0	0	0	0	0	Unknown 1
Dlo_026772.1	*DlU2*	0	50.68	397.92	1516.17	0.97	0	0	0	0	0	0	0	0.24	Unknown 2
Dlo_028569.1	*DlU3*	0	558.23	418.54	121.81	0.78	7.07	0	3.14	4.43	3.26	3.67	0	0	Unknown 3
Dlo_019949.1	*LEA5*	36,215.45	27.67	145.54	256.16	3165.31	6822.69	861.14	7685.55	11,712.56	3205.39	24,022.25	1205.56	3737.57	Late embryogenesis abundant protein 5
Dlo_008311.1	*CNOT3*	11,187.75	12.96	27.95	44.39	306.61	11,545.36	933.45	1611.48	393.14	1560.72	2650.38	7249.71	1296.04	CCR4-NOT transcription complex 3
Dlo_028175.1	*DlU4*	10,885	0.31	2.94	3.3	5220.16	576.03	3.61	217.29	35.02	4434.9	1942.11	5575.02	396.85	Unknown 4
Dlo_017033.1	*PR1–1*	5645.16	40.36	26.32	10.4	2601.85	122.2	1.45	86.79	22.85	0.96	361.66	577.38	17.99	Pathogenesis-related protein 1–1
Dlo_022694.1	*DC2.15*	3546.1	0.29	0	0	965.82	92.92	9.21	930.75	46.71	788.54	400.61	1323.15	301.29	14 kDa proline-rich protein DC2.15
Dlo_030516.1	*CHI*	3288.7	12.16	54.25	8.31	2.21	1.5	0.7	0.62	0.45	0	0.46	1.65	0.19	Chitinase
Dlo_030074.1	*PR-4*	3170.89	0.31	2.48	1.62	517.32	105.29	0.59	90.21	85.99	0.79	10.68	4.68	25.33	PR-4 protein
Dlo_028350.1	*CAT*	2411.98	15.42	41.81	55.28	4254.18	1219.17	398.73	293.83	464.27	651.57	3884.71	264.3	319.86	Catalase
Dlo_011004.1	*NsLTP2*	2367.71	0	0	0	1410.22	836.82	1.46	171.89	732.06	69.9	148.79	31.47	45.31	Non-specific lipid-transfer protein 2
Dlo_032927.1	*PIP2.1*	2191.16	8.96	7.95	4.38	2253.88	162.85	81.62	45.29	21.99	140.46	842.74	1733.29	217.06	Aquaporin PIP2.1
Dlo_011314.1	*POD P7*	2007.14	3.73	4.04	2.59	67.72	32.71	1.25	33.7	10.84	1.82	24.34	4.33	1.23	Peroxidase P7-like
Dlo_020889.1	*DlU5*	1534.05	0.27	18.34	62.55	1224.15	79.36	8.05	83.74	14.28	2100.25	1136.71	29,080.32	892.17	Unknown 5
Dlo_009170.1	*OSM1*	1459.85	8.09	7.15	4.64	1450.79	3031.81	58.61	3028.02	3208.04	144.45	3184.56	2337.21	430.64	Osmotin-like protein 1
Dlo_006330.1	*EXLB1*	1145.83	1.14	4.4	3.19	303.02	2258.39	7.29	32.98	200.08	12.5	543.39	0.26	111.72	Expansin-like B1
Dlo_003365.1	*DIR1*	1049.5	0	0	0	614.07	12.2	5.3	35.6	37.19	284.21	88.68	0.7	24.18	Lipid-transfer protein DIR1
Dlo_024175.1	*CHI*	983.75	0.14	0	1.5	3965.12	6.77	5.9	46.39	47.66	1.53	10.77	5.23	5.17	class I chitinase
Dlo_011438.1	*PIP1*	812.41	0.56	1.69	2.71	1138.83	1183.76	441.46	491.94	345.78	1651.58	806.49	239.44	651.72	Aquaporin PIP1
Dlo_014725.1	*PME1*	753.54	0	1.19	0.57	501.17	0.62	0.96	0.53	0	16.39	1.39	21.11	8.61	Pectinesterase precursor
Dlo_027164.1	*CHS*	610.74	0.1	1.76	0.91	1988.95	12.84	204.09	222.61	19.12	40.45	5.95	0.96	122.71	chalcone synthase
Dlo_023321.1	*TLP1*	573.07	0.8	0.14	0.26	39.55	75.91	0.5	162.54	45.36	2.27	2.42	0.79	0.99	thaumatin-like protein
Dlo_000377.1	*POD5*	566.61	1.32	1.3	1.41	79.76	728.88	601.84	3319.92	905.76	380.12	225.83	1440.49	117.02	Peroxidase 5
Dlo_004275.1	*TIP2–1*	546.09	0	0	0	55.57	139.05	71.7	147.25	132.03	102.82	84.76	0.26	45.97	aquaporin MIP family TIP2–1
Dlo_017039.1	*PR1-like*	462.02	7.87	3.65	2.14	52.13	26.21	0	121.87	14.14	137.08	3.17	0.42	0	Pathogenesis-related protein 1
Dlo_017003.1	*GAST-like*	459.7	0.24	5.45	5.45	28.63	219.33	1.56	1181.5	449.48	0.89	3.56	67.29	4.4	Gibberellic Acid Stimulated Transcript-like
Dlo_012593.1	*DlU6*	433.86	0	0	0	0	0	0.64	0	0.73	1.88	0.74	0	0	Unknown 6
Dlo_005429.1	*ERF114*	352.05	0	2.51	1.98	9.51	137.66	32.36	98.04	49.11	1.79	2.92	0.53	3.31	ethylene-responsive transcription factor
Dlo_032871.1	*GST*	293.99	0	0	0	0	0	0	0	0	0	2.07	0	0	glutathione S-transferase
Dlo_014570.1	*GLP3*	259.25	0.18	4.45	4.26	107.7	0.72	4.66	2.57	1.99	0	0	0	0.23	germin-like protein 3

On the contrary, 28 representative NEC marker genes were highly and preferentially expressed in NEC, barely or undetected in EC, ICpEC and GE, including *LEA5*, *CCR4-NOT transcription complex subunit 3* (*CNOT3*), *pathogenesis-related protein* (*PR1–1*, *PR1-like*, *PR4*), *14 kDa proline-rich protein DC2.15* (*DC2.15*), *chitinases* (*CHI*: Dlo_030517.1, Dlo_024175.1), *catalase* (*CAT*), *Lipid transfer proteins* (*NsLTP2*, *DIR1*), *aquaporins* (*PIP1*, *PIP2.1*, *TIP2–1*), *peroxidases* (*POD-P7*, *POD5*), *osmotin-like protein 1* (*OSM1*), *expansin-like B1* (*EXLB1*), *Pectinesterase precursor* (*PME1*), *chalcone synthase* (*CHS*), *thaumatin-like protein* (*TLP1*), *Gibberellic Acid Stimulated Transcript-like* (*GAST1*), *ethylene-responsive transcription factor 114* (*ERF114*), *glutathione S-transferase* (*GST*, Dlo_032871.1), *germin-like protein 3* (*GLP3*), and three unknown genes (*DlU4*, *DlU5*, *DlU6*) (Table 4). The NEC-specific marker genes maybe the key inhibitor of the transition from NEC to EC, while the SE markers may function on SE development.

### qRT-PCR verification of selected molecular markers

To experimentally confirm that the molecular markers were indeed expressed and played a key role during longan SE, 16 molecular markers, including 8 transcription factors *DlLEC1*_Dlo_017092.1, *DlL1L*_Dlo_020821.1, *DlABI3*_Dlo_012160.1, *DlWOX9*_ Dlo_022316.1, *DlWOX2*_Dlo_032045.1, *DlAGL80*_Dlo_017585.1, *DlBBM*_Dlo_011527.1 and *DlPLT2*_Dlo_004646.1, auxin metabolism gene *DlGH3.6*_ Dlo_020986.1, auxin polar transport gene *DlPIN1*_Dlo_020694.1, 3 meristem growth regulation genes *DlPDF1.3*_ Dlo_030812.1, *DlRGF3*_Dlo_026048.1, *DlGIF2*_Dlo_026819.1, 2 extracellular protein encoding genes *DlLTP*_Dlo_013012.1 and *DlCHI*_Dlo_030516.1, a late embryogenesis abundant protein gene *DlLEA5*_Dlo_019949.1, were selected for qRT-PCR identification in the synchronized cultures at distinct developmental stages during longan SE, including NEC, EC, ICpEC, GE, torpedo-shaped embryos (TE) and cotyledonary embryos (CE).

**Fig. 6:**
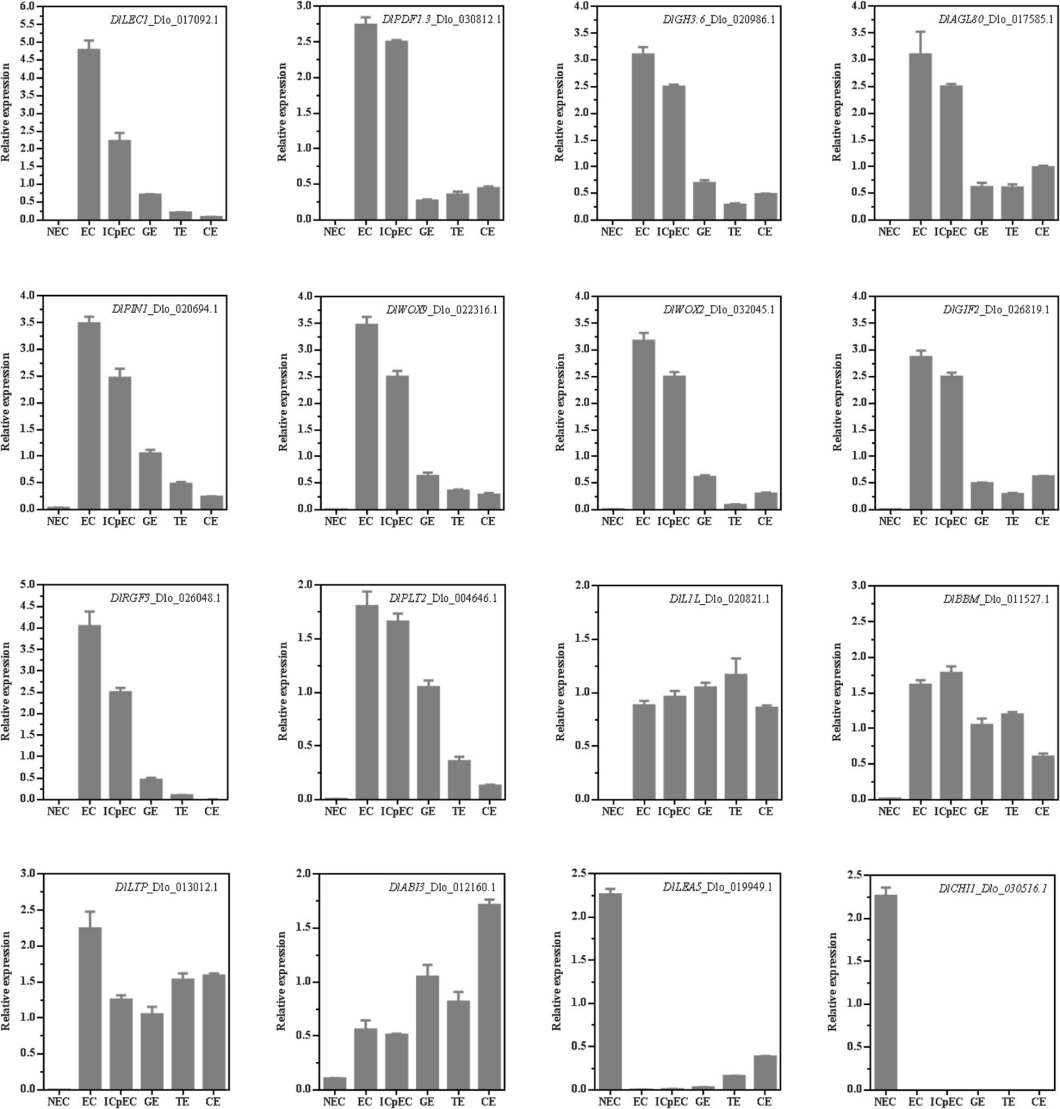
qRT-PCR verification of the selected molecular markers during longan SE. Non-embryogenesis callus (NEC), friable-embryogenesis callus (EC), incomplete compact pro-embryogenic cultures (ICpEC), globular embryos (GE), torpedo-shaped embryos (TE) and cotyledonary embryos (CE). *DlFSD*, *DlEF1a*, and *Dlelf4a* are used as reference genes. Data are means±SD (*n* = 3)

Base on the qRT-PCR results, all selected genes were expressed at varying levels at different development stages (Fig. 6). The selected molecular markers *DlLEC1*, *DlPDF1.3*, *DlGH3.6*, *DlPIN1*, *DlWOX9*, *DlWOX2*, *DlGIF2*, *DlRGF3*, *DlPLT2* and *DlAGL80* were barely or undetected in NEC, while they mainly expressed during early SE, they all highest expressed in EC and then down-regulated during SE, showed relative low expression in TE and CE, indicated that those molecular markers played an important role in EC induction and maintainance. Meanwhile, *DlL1L*, *DlBBM*, *DlABI3* and *DlLTP* were highly expressed or up-regulated during SE processes, and minimally or undiscovered expressed in NEC, suggested that those marker genes may positive regulated the longan SE development. In addition, the transcription level of *DlLEA5* and *DlCHI* were highly and specific expressed in NCE, they may the inhibitor of the transition from NEC to EC. qRT-PCR validation of SE-related genes also showed a high correlation between RNA-seq and qRT-PCR data (Additional file 10: Table S6).

## Discussion

### Auxin and cytokinin play an important role in longan SE

It is well know that auxin and cytokinin (CTK) were key factors of plant cell division, differentiation, and SE induction [59]. Meanwhile, the level of endogenous IAA and CTK were influenced by the application of exogenous auxin and CTK [3, 10, 60–62]. Auxin was consider as a central regulator in SE, probably due to the establishment of auxin gradients during SE induction [9]. So far, the exogenous application of auxin during SE has been well documented [9, 10, 27, 60]. Among the auxin, 2,4-dichlorophenoxyacetic acid (2,4-D) was most effective and widely used for induction of SE in several plants [63–65]. The level of endogenous IAA was correlated with pro-embryogenic mass formation and high-frequency SE competency [66]. Previous study had also proved that dynamic change of endogenous IAA was among the first signals leading to the induction of SE [67].

Over the past 20 years, longan SE has been established and widely used as model system for embryogenesis in woody plants, high concentration of 2,4-D in MS medium was require for inducing EC from immature zygotic embryo, while it suppressed the further development of SE, moreover, 2,4-D and KT were the key factors in long term maintenance of longan EC [1, 7, 8]. Subsequent studies revealed that controlling the doses of 2,4-D could synchronized regulated the developmental processes of longan SE, withdrawal of 2,4-D from the medium triggered further embryo development [46, 68–70]. The level of endogenous IAA and CTK in early SE stages (EC, ICpEC and GE) were much higher than NEC stage, IAA level reached the peak in GE and then significantly decreased at later stages. In addition, the level of IAA higher than CTK at the same stage during early SE. The results indicated that high level of endogenous IAA and lower level of CTK were essential for early SE [3]. However, the molecular mechanism responsible for the endogenous IAA and CTK level changing during SE, and potential crosstalk with each other or other factors remains poorly-understood.

The increase of IAA during longan early SE might be due to the increased biosynthesis and transition of endogenous auxin precursor [9]. The tryptophan (Trp) dependent IAA biosynthesis was an important pathway in higher plants, exogenous applied the doses of Trp and IAA had similar enhancement during rice SE [71]. In our study, the expression level of *ASA*, *IGS*, *TSA*, *TSB*, the key genes in Trp synthesis, were drastic up-regulated in EC and remained high in early SE, only *PAI* showed NEC specific with low FPKM, suggested that the level of Trp during early SE was higher than NEC, high IAA level might be due to high level of auxin precursor during early SE. *YUCCAs* family encoding key enzymes in IAA biosynthesis, were required for SE induction in *Arabidopsis* [72], and three *YUCCAs* and *AAO1*, one *NIT*, *CYP71A13* and three *ST5a*, showed up-regulated expression from NEC to EC, two *YUCCAs*, *AAO1*, *ST5a* were down-regulated during early SE, while *YUCCA*_Dlo_013505.1 kept up-regulated expression during early SE. Other IAA synthesis genes were mainly expressed in NEC with minimal FPKM. The increase of IAA level may due to these differentially expressed genes during early SE. However, more evidences is needed to prove the relationship between these DEGs and increased IAA level.

During SE induction of *C. canephora*, the balance of free IAA and IAA conjugates was essential for embryogenic potential [73], the conjugation of auxin was synthesized by *GH3* family [74], we found that *GH3* family genes were minimal expressed in NEC, most of them dramatic up-regulated in EC and down-regulated during early SE, indicated that the conjugated IAA also played an important role in longan SE. Previous studies had revealed that auxin transports was complex and highly regulated for embryogenic development [75]. *TIR1* mediated Aux/IAA proteins degradation and auxin-regulated transcription in the present of auxin [76], while *TIR1* genes were down-regulated in EC and remained low during longan early SE. *AUX1*, which mediated influx of IAA into cells, were mainly expressed in NEC, and down-regulated during early SE. *PIN1* played a fundamental role in maintaining the embryonic auxin gradients [77], were up-regulated in EC and kept high in ICpEC and GE in our study.

Auxin transcriptionally activated *Aux/IAA*, *GH3* and *SAUR* family, the *Aux/IAA* family had 29 members in *Arabidopsis*, but not all members were induce by auxin [78]. *SAUR* was the most abundant family of early auxin-inducible genes, but only few members had been functional characterized, *OsSAUR39* was reported to negatively regulate auxin biosynthesis and transport [79]. *ARF* showed strongly disturbance during zygotic embryo development [80, 81], and *ARF5* seemed to be importance for SE [82]. Further transcript analysis during SE revealed that the components of auxin signaling: *Aux/IAA*, *ARF*, *SAUR* and other auxin-responsive genes were wildly modulated during SE [9, 13]. In our study, 11 *ARF* significantly up-regulated in EC and remained high during early SE, three *ARF* first down-regulated in EC and then up-regulated during SE. *IAA* family showed the similar expression pattern during SE, while most of *SAUR* were mainly expressed in NEC.

Other than auxin being a main inducer of SE, exogenously supplied CTK to induce SE was well established in a lot of species [83–85]. Large numbers of transcripts involved in zeatin biosynthesis and signal transduction were differentially expressed during cotton SE [10]. Meanwhile, endogenous CTK level were higher in SE than in NEC [3]. From NEC to EC, a total of 40 DEGs implicated in cytokinin signal transduction, including 22 *CRE1* (seven up-regulated and 15 down-regulated), 15 *B-ARR* (five up-regulated and 10 down-regulated), 3 *A-ARR* (one up-regulated and two down-regulated). During early SE, a total of 32 DEGs involved in cytokinin signaling pathway, most of them were up-regulated during early SE. In zeatin synthesis pathway, *TRIT1* was up-regulated from NEC to ICpEC, most of *CisZOG*, *IPT1,4*, *CYP735A*, *CKX*, *UGT76C* were down-regulated in EC and remained low during early SE. However, IAA and zeatin biosynthesis and signal transduction related genes showed complex and integrated regulation during SE, further study of these genes is required in longan SE.

### SE-related molecular marker genes play a key role during longan SE

The molecular marker genes for longan SE belong to several distinct functional categories, they can be used to mark the embryogenic potential of plant cells and study various biochemical and physiological processes of plant embryogenesis and development. A number of transcription factors (TFs) had been reported as key factors in SE induction. In *Brassica napus*, *LEC1*, *LEC2*, *FUS3*, *ABI3*, *WOX9*, *WOX2*, *BBM*, genes belonged to TFs, were identified as molecular markers for early microspore embryogenesis [86]. In our study, 10 molecular markers were TFs (*DlLEC1*, *DlL1L*, *DlLEC2*, *DlABI3*, *DlFUS3*, *DlWOX9*, *DlWOX2*, *DlAGL80*, *DlBBM*, *DlPLT2*), their functions on embryogenesis had been well characterized in various plants. Ectopic expression of *LEC1* was sufficient to trigger embryogenic potential and to induce somatic embryo from *Arabidopsis* leaf surface [87]. *ZmLEC1* was used as a reliable marker for early SE in maize as its expression pattern during SE was similar to that of *AtLEC1* during zygotic embryogenesis [88]. Mutational analyses in *Arabidopsis* showed that *LEC* genes were essential for induction of SE [37]. Ectopic expression of a carrot *C-LEC1* which was driven by *AtLEC1* promoter, rescued the defects of *lec1–1* mutant [89]. Moreover, ectopic-expressed *AtLEC1* in tobacco induced the start of embryogenic transition [90]. The *LEC1-like (L1L)* was most closely related to *LEC1* and required for normal embryo development, ectopic-expressed *L1L* in *Arabidopsis* can complement *LEC1* functions [91]. Meanwhile, *L1L* expression was mainly accumulated in the early stage SE of *Theobroma cacao* [92], *Vitis vinifera* [93], and *Helianthus annuus* [94].

*LEC2*, *ABI3*, *FUS3* were B3 domain-containing transcription factors, ectopically expressed *AtABI3* do not induced SE but endowed the embryo with traits to seedling [95]. *BBM* and *PLT2* were clustered to AP2/ERF transcription factor family, their functions on embryogenesis and root meristem were overlap [96–98]. Over-expression of *BBM* triggered spontaneous somatic embryo formation in *Arabidopsis thaliana* and *Brassica napus*, *BBM* was server as a marker for embryogenesis cells in *Brassica napus* [96]. Recently study show that *BBM* and *PLT2* induced SE in a quantitatively and context dependent manner by LEC1-ABI3-FUS3-LEC2 (LAFL) network, and *LAFL*/*AGL15* were required for *BBM* mediated embryogenesis [40]. In this assay, *DlLEC1*, *DlLEC2*, *DlFUS3* were early SE-specific genes, *DlL1L*, *DlBBM*, *DlABI3*, *DlPLT2* were highly expressed during the SE processes, they can be used as remarkable markers for longan early SE.. To date, *AGL15* was the only MADS-BOX member which preferentially expressed in developing embryos and promote the initiation of SE [44, 45], and *AGL80* was essential for the central cell and endosperm development [99]. However, *DlAGL15* was considered as poor marker. Firstly, we suggested another MADS-BOX gene *DlAGL80*, a SE-specific gene as a new marker for longan early SE.

*WUS* was a critical regulator for stem cell fate in the shoot apical meristem [100]. Over-expression of *AtWUS* initiated the acquisition of embryogenic competence in *Gossypium hirsutum* [41, 42]. *WUS* was suggested as a useful gene marker for SE initiation [101]. Meanwhile, *WOX* genes marked cell fate during early embryogenesis in *Arabidopsis* [102], *WOX2* was used as potential marker during early SE [103]. *STIMPY****/****WOX9* played an important role in promoting cell proliferation and preventing precocious differentiation in emerging seedlings [104]. *WOX2* and *WOX9* were highly expressed at the early stage of SE in *Picea abies*, they may function together on conifer embryo patterning [105]. In addition, *DlWUS* was isolated from embryogenic callus and expressed in all the stage of SE, which consistent with our transcriptome date suggested that *DlWUS* genes were poor markers during longan SE. qRT-PCR verification demonstrated that *DlWOX2* and *DlWOX9* were specific expressed in early SE and down-regulated during SE, they might played an importance role in longan early SE.

*PIN1* was involved in auxin polar transport and cellular differentiation during embryogenesis [106, 107]. Antisense expression of *PIN1* disrupted the formation of somatic embryos and reduced the expression of SE-related genes, indicated that *PIN1* was essential for SE induction [108]. *GH3.6* was contribute to maintain auxin homeostasis by converting excess IAA to IAA-amino acid conjugates, over-expression of *GH3.6* significantly enhanced the accumulation of IAA-Asp [109]. *DlPIN1* and *DlGH3.6* were specific expressed in early SE and down-regulated during SE.

In our study, *CYP78A5* and *CYP87A3* were most abundant in EC and follow by ICpEC stage. In *B. napus*, *CYP78A5* was identified as an early marker for microspore-derived embryos development [86]. *PDF1.3* was closely related to Arabidopsis *Protodermal factor 1*, a gene exclusively expressed in L1 layer of vegetative, inflorescence, floral meristems and specific-expressed in protodermal cell during embryogenesis which related to cell fate determination [110]. In addition, *AtGRP-5* was associated with somatic embryo formation in Arabidopsis and eggplant [111]. *RGF3* and *GIF2* were key genes of cell proliferation, showed SE-specific expression pattern during early SE. *RGF3* belonged to *root meristem growth factors* family that played the redundant role in maintaining the post-embryonic root stem cell niche and by positive regulating cell proliferation [112]. *GIF2* was required for cell proliferation and lateral organs grow [113]. Those SE-related genes *DlPIN1*, *DlGH3.6*, *DlPDF1.3*, *DlGRP-1*, *DlRGF3* and *DlGIF2* can be use to mark the early stage of longan early SE. Furthermore, *DlLBP*, *DlKCS*, *DlZDS* and *DlRPL17*, *DlMAN7* and *DlU1*, *DlU2*, *DlU3* were specific accumulated in early SE, despite that no functions on SE have been published yet for them, suggested that they might be the key genes for longan SE.

*SERK* played a key role in the acquisition of embryogenic competence in plant cells, *DcSERK* was identified as a suitable marker for SE as it only abundant in embryogenic cultures and ceased after the globular stage, but not in any other tissues [34]. In *Dactylis glomerata*, *SERK* showed the similar expression pattern with *DcSERK* and used as a convenient marker for cells competent to form embryos in monocots [114]. *AtSERK1* was highly expressed during *Arabidopsis* embryogenic cell formation and early embryogenesis, suggested that *AtSERK1* was sufficient to mark embryogenic competence in culture [115]. However, *ZmSERKs* were detected in non-embryogenic callus [116], and the identification of *SERK* genes in rice [117], and wheat [118], suggested that their functions were not limit to embryogenesis. In our study, *DlSERKs* were expressed not only in SE stages, but also in non-embryogenic callus and other tissues.

*LEA5* belonged to the fifth group of late embryogenesis abundant proteins gene, were abundant in late embryogenesis of mature seed, and involved in the abiotic stresses responses [119]. *CNOT3*, might be a new *CCR4-NOT complex* gene in plant, which had proved in regulation of cell division in HeLa cell, while its functions on plant was poorly-understood. *14 kDa proline-rich protein DC2.15* was connected with the initiation of embryogenesis by the removal of auxin [120]. Aquaporins were the major channels of water transport pass through biological membranes, and involved in cell expansion, organ movement and elongation [121]. It is widely acceptable that the extracellular proteins (such as GLPs, LTPs, CHIs) were required for plant differentiation and morphogenesis, they were used as protein markers for SE [58]. Our study revealed that a total of 28 transcripts were specific and extreme-highly expressed in NEC, while barely or undetected during early SE stages, for example, the *LEA5*, *CNOT3*, *DC2.15*, *PIP1;2*, *PIP2;1*, *GLP3*, *NsLPT*, *CAT*, *POD*, *GST*, et al., these genes might play an important role in the transition from NEC to EC. However, some of these markers belonged to the certain gene family with distinct expression patterns during SE, further study of these genes function on longan SE is required.

## Conclusions

In summary, our study generated a high resolution transcriptome datasets for longan SE. A comparative analysis of global gene expression patterns during early SE stages provided subsets of DEGs that regulated SE in longan. Our study revealed the expression profiles of genes involved in plant hormones such as auxin and cytokinin signaling pathway, flavonoid and fatty acid biosynthesis pathway, extracellular protein, as well as the representative molecular marker genes, indicating their possible roles in longan SE. This transcriptomic data provides new insights into future functional studies, as a means of studying the molecular mechanisms in SE.

## Materials and methods

### Plant material and RNA extraction

The synchronized cultures at different developmental stages, including non-embryogenic callus (NEC), friable-embryogenic callus (EC), incomplete compact pro-embryogenic cultures (ICpEC), globular embryos (GE), torpedo-shaped embryos (TE) and cotyledonary embryos (CE) of *D. longan* ‘Honghezi’ were obtained following previously methods [1, 7, 8, 68]. To obtained.the synchronized cultures of NEC, EC, ICpEC, GE and TE, embryogenic calli was transferred to MS basal medium (2% sucrose and 6 g/L agar, pH 5.8) supplemented with 0.2% activated carbon and 1.0 mg/L, 0.5 mg/L, 0.1 mg/L, 0.05 mg/L 2,4-dichlorophenoxyacetic acid (2,4-D), respectively. Embryogenic calli was transferred to MS basal medium (5% sucrose and 6 g/L agar, pH 5.8) to obtained the synchronized cultures of CE. The synchronized cultures of different stage were cultured in three biological replicates, each replicates consisting of 10 culture bottles, and were confirmed by the histological observations as shown schematically in Fig. 1. We collected the NEC, EC, ICpEC and GE samples from 5 bottles in each of the three replicates, and stored at − 80 °C for RNA extraction.

Total RNA was extracted separately from NEC, EC, ICpEC and GE in the three biological replicates using Trizol Reagent (Invitrogen, USA), then DNase I was used to digest any genomic DNA. Extracted RNAs were quantified by Agilent 2100 bioanalyzer (Agilent Technologies, USA) and evaluated the integrality by denaturing agarose gel electrophoresis and ethidium bromide staining. RNA samples with A260/A280 ratios between 1.9~2.1, 28S/18S ratios ≥1.0, and integrity numbers (RINs) more than 8.5 were selected to construct cDNA libraries. The RNA of the three biological replicates were mixed in equal amounts and used for cDNA library construction.

### Library construction and RNA sequencing

After purification with oligo (dT)_25_-attached magnetic beads, the mRNA was interrupted into short fragments by divalent cations under elevated temperature. Then, these cleaved RNA fragments were used to synthesize first-strand cDNA using a random hexamer primer and the SuperScript III (Invitrogen, USA) reverse transcriptase. The second-strand cDNA was subsequently synthesized using random primers and end repaired, then adaptors were ligated by T4 DNA ligase after adenylation at the 3′-end. Eventually, suitable adaptor-ligated fragments were selected as templates for PCR amplification to generate the final cDNA library. The four resulting cDNA libraries were quantified by Agilent 2100 Bioanalyzer (Agilent Technologies, Palo Alto, CA, USA) and qRT-PCR (ABI StepOnePlus Real-Time PCR System, USA), and then RNA Sequencing (RNA-seq) was carried out with an Illumina HiSeq™ 2000 system at The Beijing Genomics Institute (BGI, Shenzhen, China). The entire set of raw reads was submitted to NCBI Sequence Read Archive under the accession number: PRJNA565345.

### RNA-Seq reads mapping and differential expression

The raw reads were cleaned by removing adapter reads, reads containing poly-N larger than 10%, and low quality reads (Q_Phred_ < 20). Cleaned reads were then aligned to the longan reference genome using Bowtie software (http://bowtie-bio.sourceforge.net/index.shtml) and TopHat2 (http://ccb.jhu.edu/software/tophat/index.shtml), read count for each gene was then obtained after mapping. Gene expression levels for each sample were estimated by *RSEM* (RNA-Seq by Expectation Maximization) software [122]. The expression levels of matched genes in each cDNA library were derived and normalized to FPKM (Fragments Per Kilobase of exon per Million fragments mapped) [123]. The differentially expression analysis of the pairwise comparison of RNA-Seq libraries were confirmed by using the Poisson Distribution analysis method [124], and the False Discovery Rate (FDR) was used to determine the *P* value threshold. The unique reads with the absolute value of log_2_^(Fold_Change)^ ≥ 1 and the FDR < 0.001 were used as the thresholds to define as differentially expressed genes (DEGs) in the pairwise comparisons (NEC_*vs*_EC, EC_*vs*_ICpEC, EC_*vs*_GE, ICpEC_*vs*_GE).

### Expression annotation and functional analysis of DEGs

Gene function was annotated based on the databases of Blast Nr (NCBI non-redundant protein sequences), GO (Gene Ontology), and KEGG (Kyoto Encyclopedia of Genes and Genomes database). The GO and KEGG functional enrichment analysis of DEGs were performed to identify which DEGs were significantly enriched in GO terms or KEGG pathways. GO terms with corrected *P*-value ≤0.05 were considered as significantly enriched terms. The KEGG enrichment was determined by Rich factor, Q-value, and the number of enriched genes in this pathway. Q-value ≤0.05 was defined as those with genes that showed significant differential expression.

### Quantitative real-time PCR analysis

For qRT-PCR validation, 500 ng total RNA extracted from each stage of longan SE (NEC, EC, ICpEC, GE, TE and CE) were transcribed into cDNA with random primers and Oligo dT primer using the SYBR Ex Script™ kit (Takara, China), Sixteen unique transcripts with potential roles in longan SE were chosen and their specific primers were designed using DNAMAN 7.0. qRT-PCR was performed on the LightCycler 480 instrument (Roche Applied Science, Switzerland) in a total volume of 20 μL in each well containing 10 μL of 2× SYBR Premix Ex Taq™; 0.8 μL of each specific primer (100 nM); 1.0 μL of cDNA template (in a 1:10 dilution); and 7.4 μL of ddH_2_O. The PCR conditions were: denaturation for 60 s at 95 °C, and then 40 cycles of 10 s at 95 °C and 20 s between 58 °C and 61 °C in function of the Tm of the primers. Primer annealing specificity was examined and verified by melting curve analysis. Four-point standard curves of a fivefold dilution series (1:5 to 1:625) from pooled cDNA were used to calculate PCR efficiency. The reactions were performed in 96-well PCR plates and each experiment consisted of three biological replicates. The expressive abundance of the sixteen selected genes were calculated relative to the expression of reference genes *DlFSD*, *DlEF-1a*, and *Dlelf4a*. Data were further processed in MS Excel. Gene names, primer sequences, product sizes, and annealing temperatures are given in Additional file 11: Table S7.

## Supplementary information

**Additional file 1: Figure S1.** Gene Ontology functional classification for the pairwise comparisons of NEC_*vs*_EC, EC_*vs*_ICpEC, EC_*vs*_GE, and ICpEC_*vs*_GE.

**Additional file 2: Figure S2.** Statistic of KEGG pathway enrichment for the pairwise comparisons of NEC_*vs*_EC, EC_*vs*_ICpEC, EC_*vs*_GE, and ICpEC_*vs*_GE**. (DOC 230 kb)**

**Additional file 3: Figure S3.** Plant hormone signal transduction pathway in the comparison of NEC_*vs*_EC. Red frame represents a transcript with increased levels of expression and green frame represents transcripts with decreased levels of expression. The image was obtained from http://www.genome.jp/kegg/.

**Additional file 4: Figure S4.** Heatmap of the differentially expressed genes in plant hormone signal transduction during longan SE. (a). Abscisic acid signal transduction; (b). Gibberellin signal transduction; (c). Ethylene signal transduction; (d). Salicylic acid signal transduction; (e). Brassinolide signal transduction; (f). Jasmonic acid signal transduction. Heatmaps indicate the gene expression levels by Log2^[FPKM + 1]^ with a rainbow color scale. The IDs and names of selected DEGs are indicated to the right of the histograms.

**Additional file 5: Table S1.** Differentially expressed genes involved in plant hormones signaling pathway during longan SE. S1-a: Auxin signaling pathway; S1-b: Cytokinin signaling pathway; S1-c: Abscisic acid signal transduction; S1-d: Gibberellin signal transduction; S1-e: Ethylene signal transduction; S1-f: Salicylic acid signal transduction; S1-g: Jasmonic acid signal transduction; S1-h: Brassinosteroid signal transduction. (XLS 151 kb)

**Additional file 6: Table S2.** Differentially expressed genes involved in flavonoid biosynthesis. (XLS 50 kb)

**Additional file 7: Table S3.** Differentially expressed genes involved in fatty acid biosynthesis during longan SE. (XLS 26 kb)

**Additional file 8: Table S4.** The distinct expression pattern of extracellular protein encoding genes during longan SE.

**Additional file 9: Table S5.** The expression pattern of molecular marker genes during longan SE.

**Additional file 10: Table S6.** Comparing differential expression genes from RNA-seq and qRT-PCR during longan SE.

**Additional file 11: Table S7.** Primers used for real-time quantitative PCR.

## Data Availability

The Illumina sequence data from this study have been submitted to the NCBI sequence read archive under the accession number [PRJNA565345]. All the supporting data are included in Additional files.

## Abbreviations

*ABI3*: *Abscisic acid insensitive 3*
*AGL80*: *Agamous-like 80*
ARF: Auxin response factor
*BBM*: Babyboom
CE: Cotyledonary embryos
*CHI*: *Chitinase*
DEGs: Differentially expressed genes
*Dl*: *Dimocarpus longan*
EC: Embryogenic callus
FDR: False discovery rate
FPKM: Fragments Per Kilo-base of exon per Million fragments mapped
*FSD*: *Fe-SOD*
*FUS3*: *FUSCA3*
GE: Globular embryos
*GH3.6*: *Indole-3-acetic acid-amido synthetase*
*GLP*: *Germine-like protein*
GO: Gene Ontology
*GST*: *Glutathione S-transferase*
ICpEC: Incomplete compact pro-embryogenic cultures
KEGG: Kyoto Encyclopedia of Genes and Genomes
*LEA5*: *Late embryogenesis abundant protein 5*
*LEC*: *Leafy cotyledon*
*LTP*: *Lipid transfer protein*
NEC: Non-embryogenic callus
*PDF1.3*: *Protodermal factor 1*
*PIN1*: *PIN-FORMED1*
qRT-PCR: Quantitative real-time PCR
RNA-seq: RNA sequencing
SE: Somatic embryogenesis
TE: Torpedo-shaped embryos
*WOX*: *WUSCHEL-related homeobox*
