# Supplementary material for: Global scale transcriptome analysis reveals differentially expressed genes involve in early somatic embryogenesis in Dimocarpus longan Lour
Source: BMC Genomics. 2020 Jan 2;21:4. doi: 10.1186/s12864-019-6393-7 (PMC6941269; doi:10.1186/s12864-019-6393-7)
Supplement: Supplementary file 1 — Additional file 1: Figure S1. Gene Ontology functional classification for the pairwise comparisons of NEC_vs_EC, EC_vs_ICpEC, EC_vs_GE, and ICpEC_vs_GE. [file 12864_2019_6393_MOESM1_ESM.doc]

Figure S1 Gene Ontology (GO) functional classification for the pairwise comparisons of NEC_*vs*_EC, EC_*vs*_ICpEC, EC_*vs*_GE, and ICpEC_*vs*_GE.

**
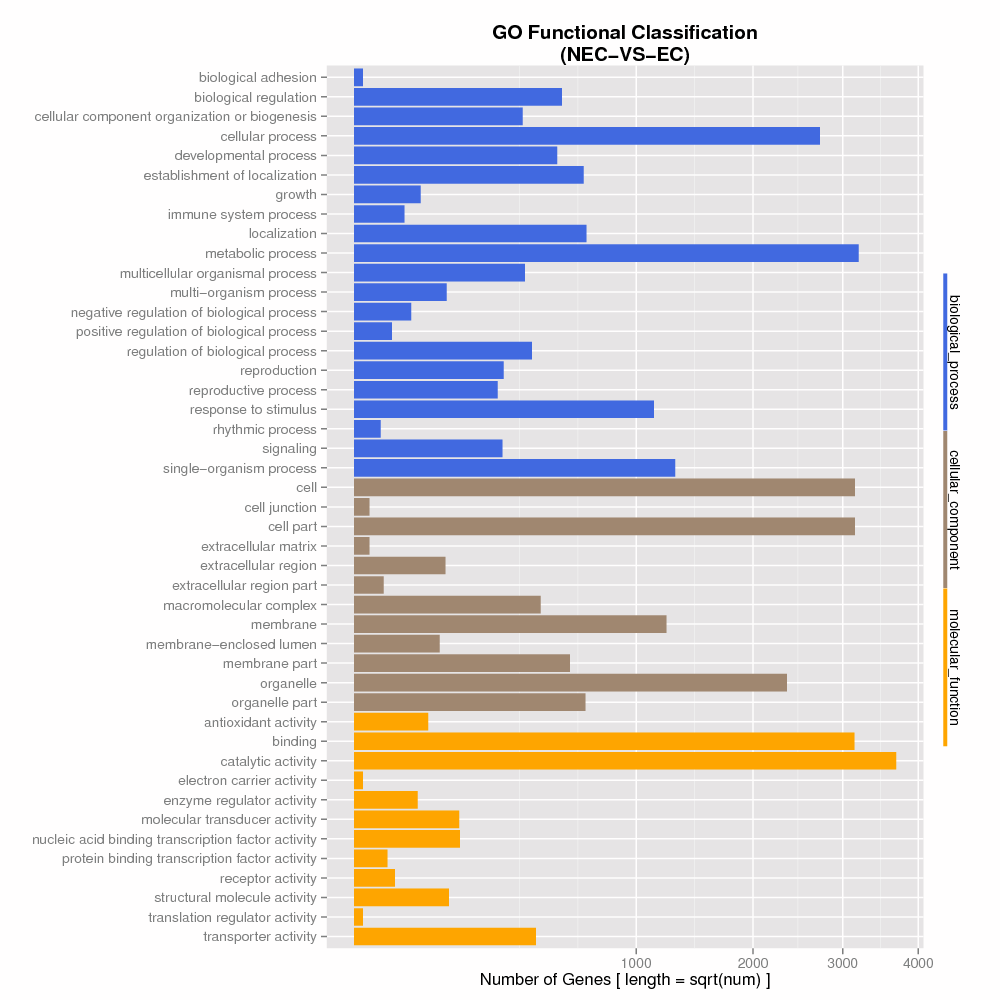

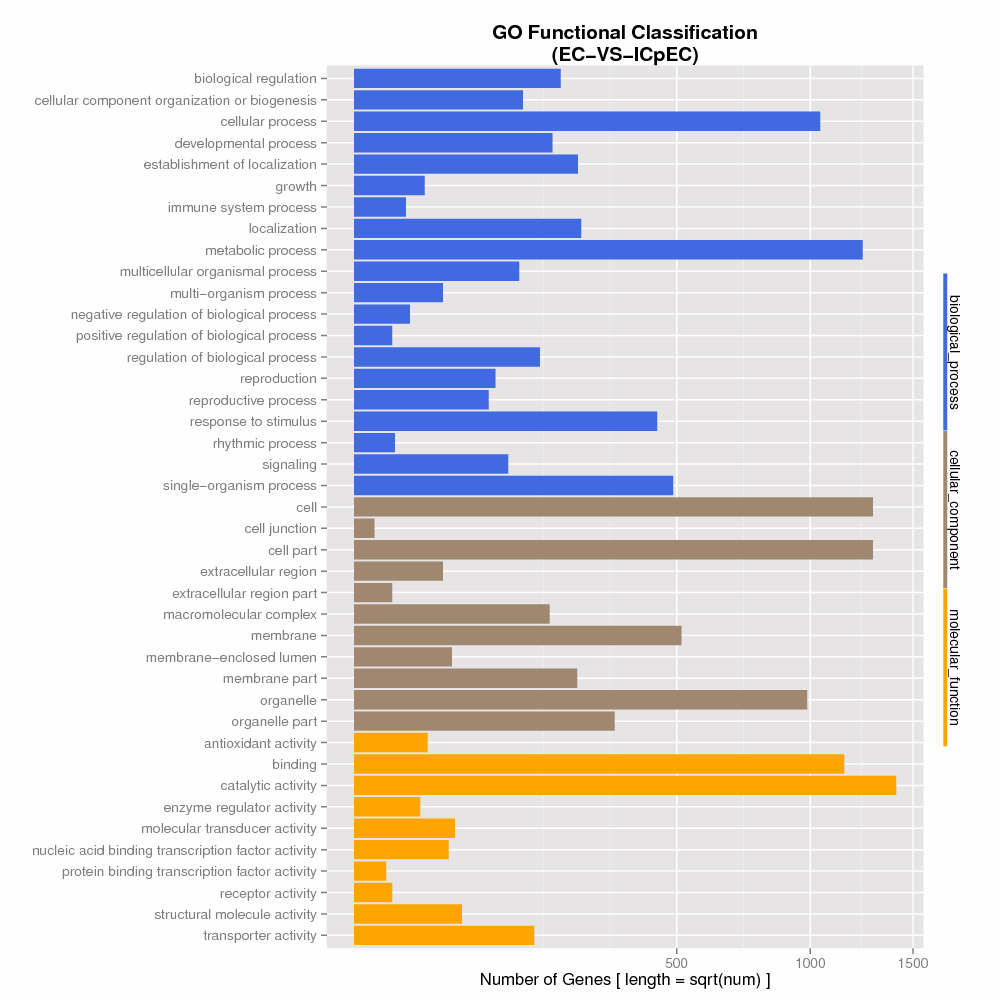
**

**
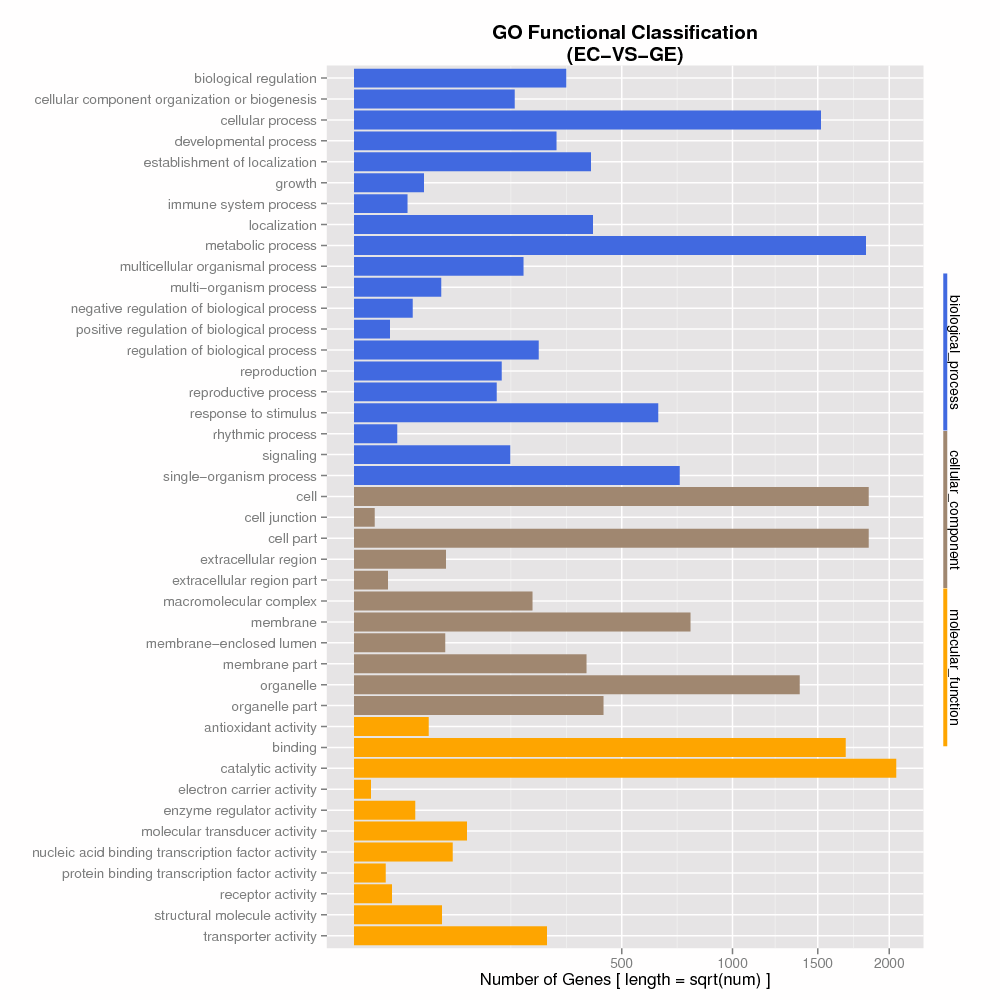
** **
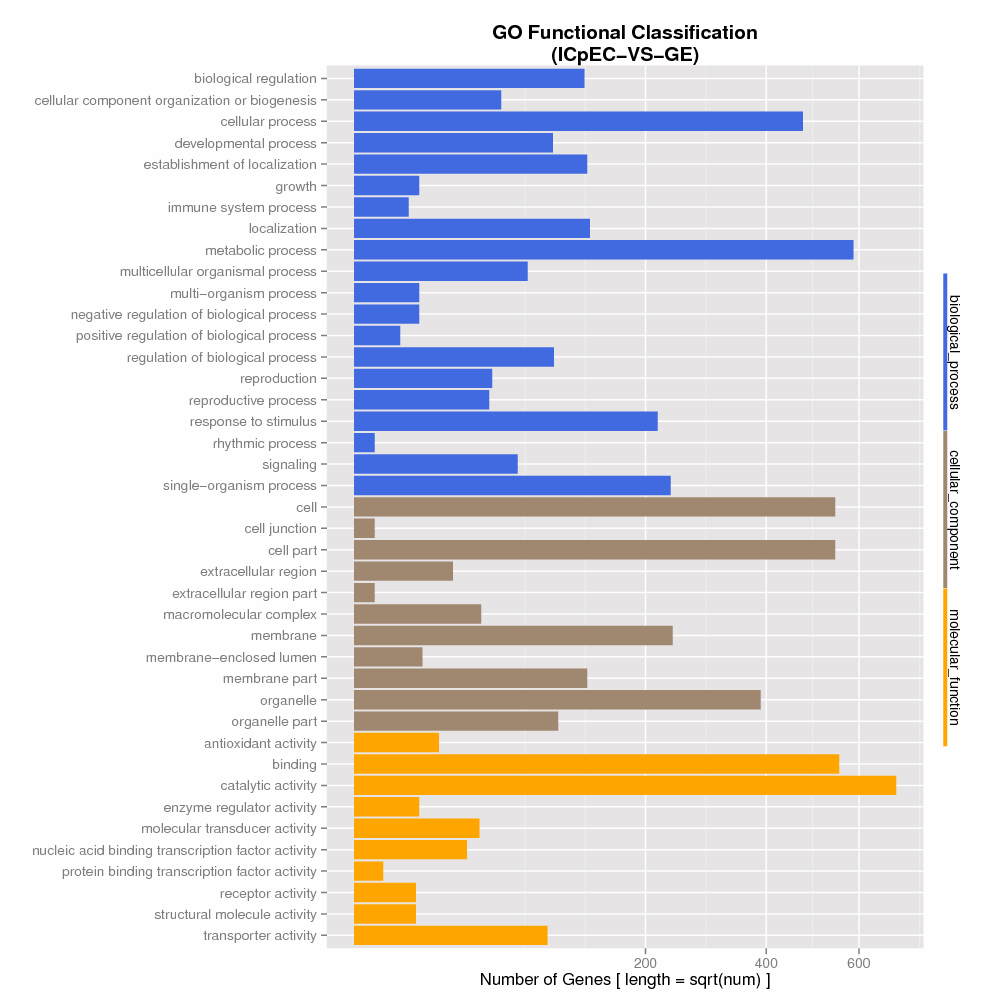
**
