# Supplementary material for: Global scale transcriptome analysis reveals differentially expressed genes involve in early somatic embryogenesis in Dimocarpus longan Lour
Source: BMC Genomics. 2020 Jan 2;21:4. doi: 10.1186/s12864-019-6393-7 (PMC6941269; doi:10.1186/s12864-019-6393-7)
Supplement: Supplementary file 2 — Additional file 2: Figure S2. Statistic of KEGG pathway enrichment for the pairwise comparisons of NEC_vs_EC, EC_vs_ICpEC, EC_vs_GE, and ICpEC_vs_GE. (DOC 230 kb) [file 12864_2019_6393_MOESM2_ESM.doc]

Figure S2 Statistic of KEGG pathway enrichment for the pairwise comparisons of NEC_*vs*_EC, EC_*vs*_ICpEC, EC_*vs*_GE, and ICpEC_*vs*_GE**.**

**
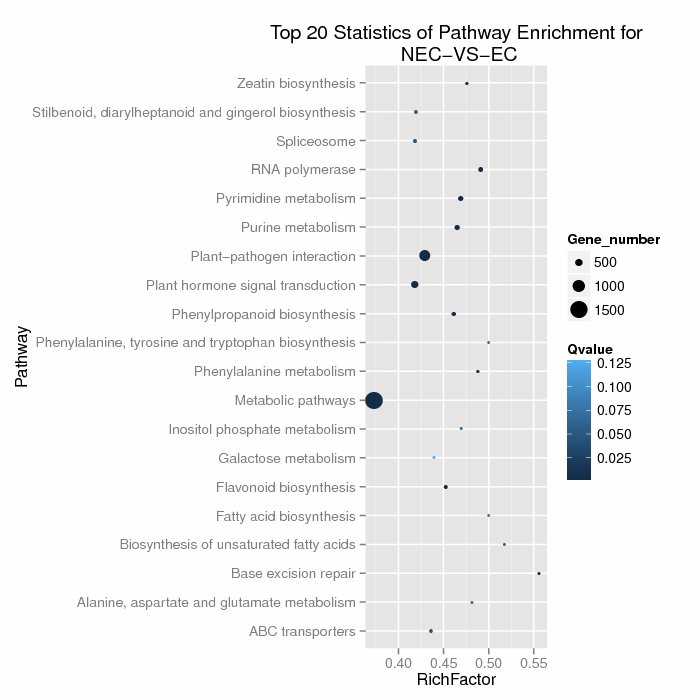

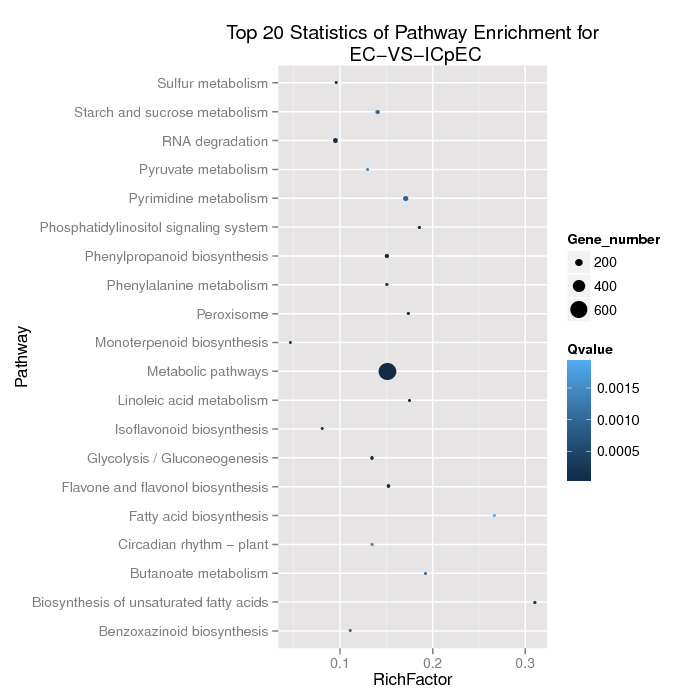
**

**
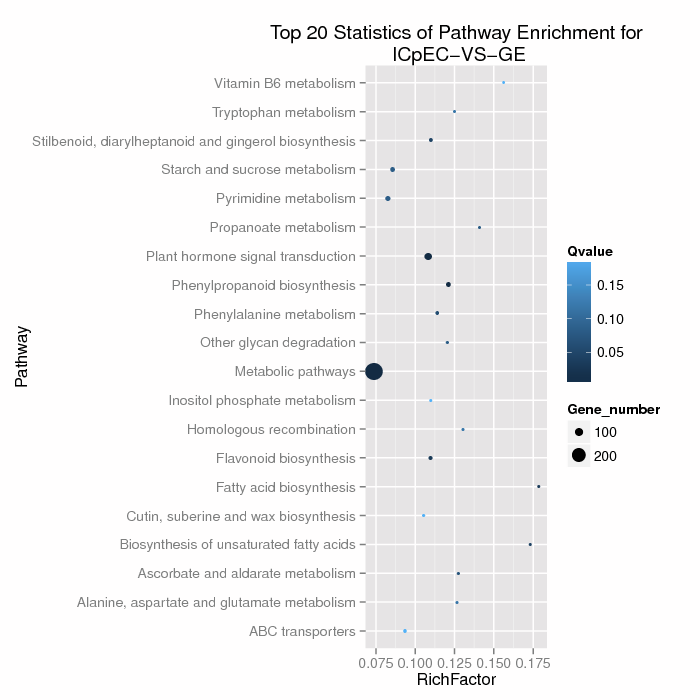

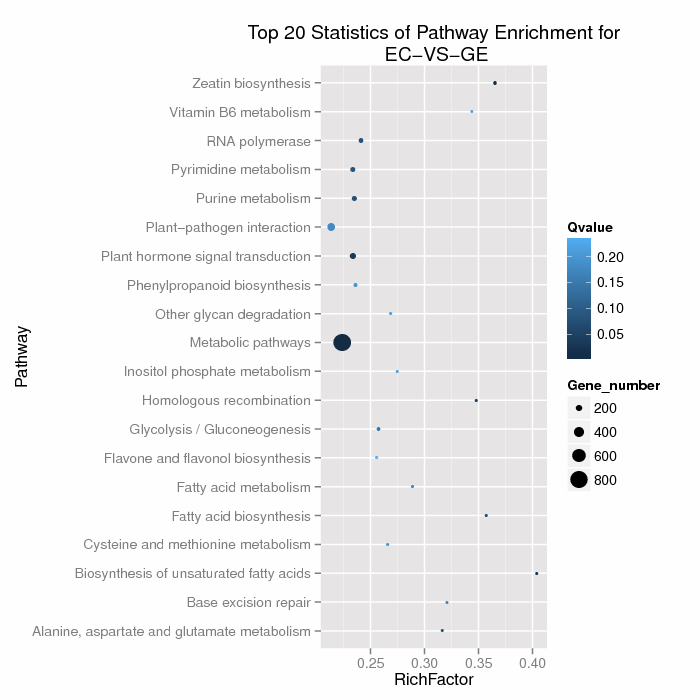
**
