# Supplementary material for: Global scale transcriptome analysis reveals differentially expressed genes involve in early somatic embryogenesis in Dimocarpus longan Lour
Source: BMC Genomics. 2020 Jan 2;21:4. doi: 10.1186/s12864-019-6393-7 (PMC6941269; doi:10.1186/s12864-019-6393-7)
Supplement: Supplementary file 3 — Additional file 3: Figure S3. Plant hormone signal transduction pathway in the comparison of NEC_vs_EC. Red frame represents a transcript with increased levels of expression and green frame represents transcripts with decreased levels of expression. The image was obtained from http://www.genome.jp/kegg/. [file 12864_2019_6393_MOESM3_ESM.doc]

Figure S3 Plant hormone signal transduction pathway in the comparision of NEC_*vs*_EC. Red frame represents a transcript with increased levels of expression and green frame represents transcripts with decreased levels of expression. The image was obtained from <http://www.genome.jp/kegg/>.

**
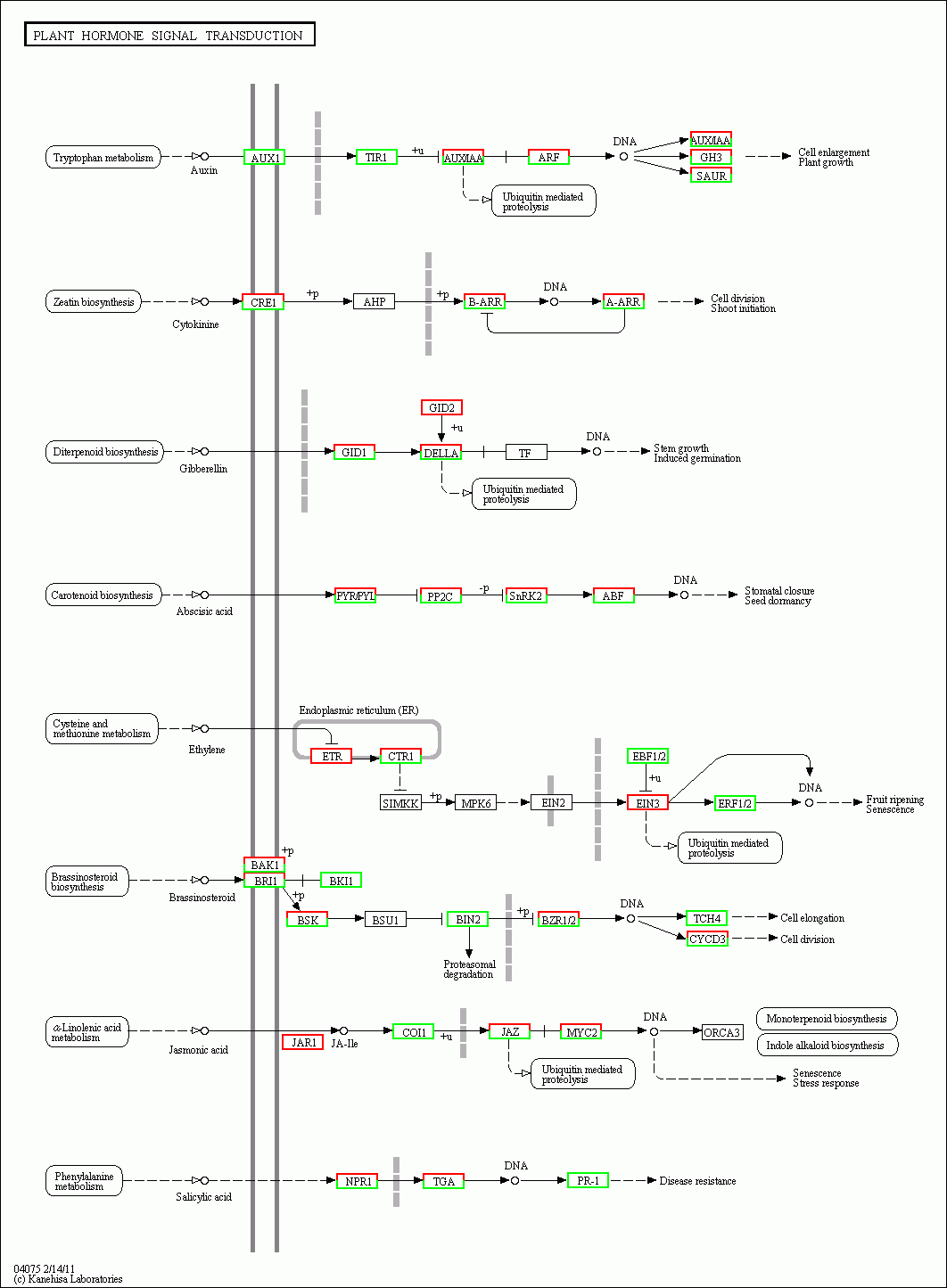
**
