# Supplementary material for: Global scale transcriptome analysis reveals differentially expressed genes involve in early somatic embryogenesis in Dimocarpus longan Lour
Source: BMC Genomics. 2020 Jan 2;21:4. doi: 10.1186/s12864-019-6393-7 (PMC6941269; doi:10.1186/s12864-019-6393-7)
Supplement: Supplementary file 4 — Additional file 4: Figure S4. Heatmap of the differentially expressed genes in plant hormone signal transduction during longan SE. (a). Abscisic acid signal transduction; (b). Gibberellin signal transduction; (c). Ethylene signal transduction; (d). Salicylic acid signal transduction; (e). Brassinolide signal transduction; (f). Jasmonic acid signal transduction. Heatmaps indicate the gene expression levels by Log2[FPKM + 1] with a rainbow color scale. The IDs and names of selected DEGs are indicated to the right of the histograms. [file 12864_2019_6393_MOESM4_ESM.doc]

Figure S4 Heatmap of the differentially expressed genes in plant hormone signal transduction during longan SE. (a). Abscisic acid signal transduction; (b). Gibberellin signal transduction; (c). Ethylene signal transduction; (d). Salicylic acid signal transduction; (e). Brassinolide signal transduction; (f). Jasmonic acid signal transduction. Heatmaps indicate the gene expression levels **by Log2^[FPKM+1]^** with a rainbow color scale. The IDs and names of selected DEGs are indicated to the right of the histograms.

| **(a)**  **(c)**  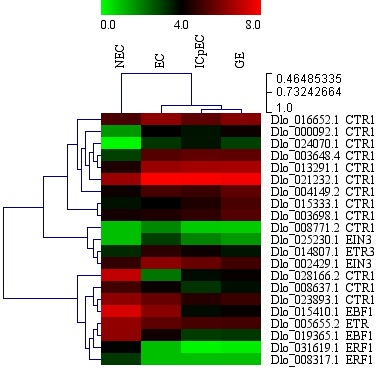  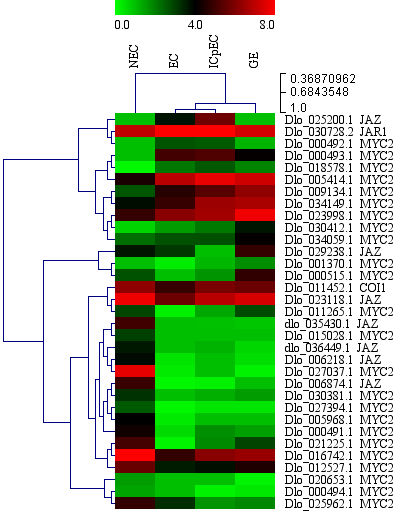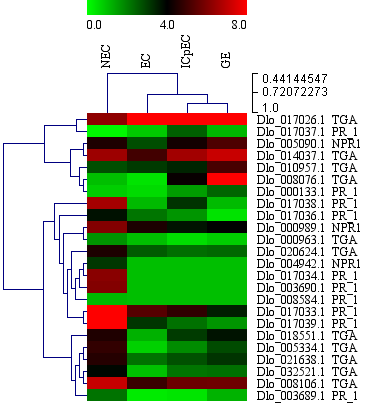  **(d)**  **(f)**  **(e)**  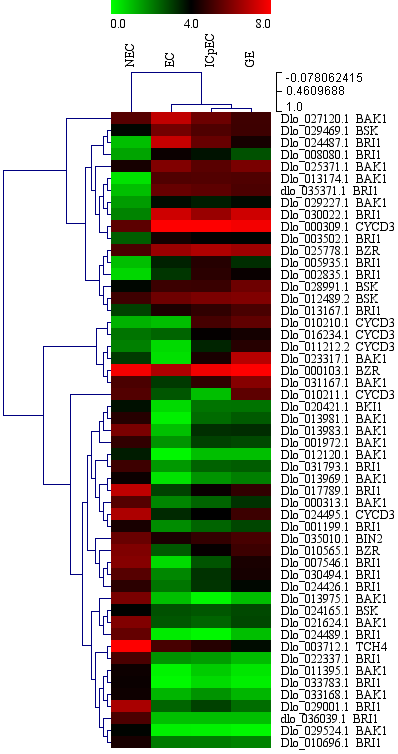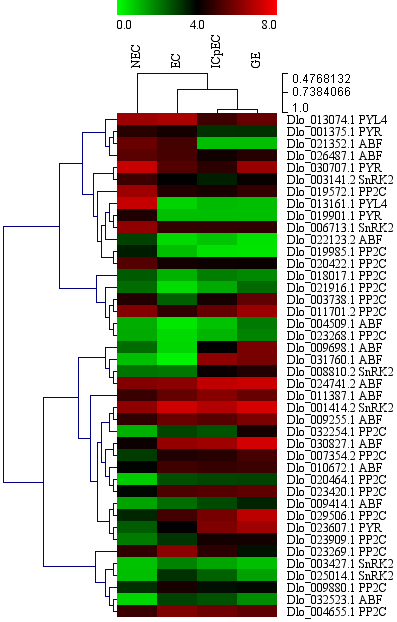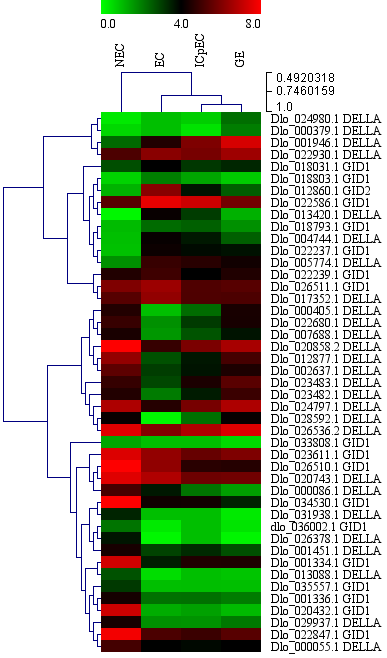  **(b)** |
| --- |
