# Supplementary material for: Global scale transcriptome analysis reveals differentially expressed genes involve in early somatic embryogenesis in Dimocarpus longan Lour
Source: BMC Genomics. 2020 Jan 2;21:4. doi: 10.1186/s12864-019-6393-7 (PMC6941269; doi:10.1186/s12864-019-6393-7)
Supplement: Supplementary file 11 — Additional file 11: Table S7. Primers used for real-time quantitative PCR. [file 12864_2019_6393_MOESM11_ESM.doc]

Table S7 Primers used for real-time quantitative PCR.

| Gene_id | Perimer | Primer sequence (5' to 3') | Product size (bp) | Annealing temperatures (^0^C) |
| --- | --- | --- | --- | --- |
| Dlo_017092.1 | *DlLEC1*-q-F | AACGCAAGACCATCACCTC | 182 | 60 |
|  | *DlLEC1-*q-R | AACCTTACCACCATCACCG |  |  |
| Dlo_020821.1 | *DlL1L-*q-F | CACGGTGAGGGAACAAGA | 99 | 58 |
|  | *DlL1L-*q-R | TTGGCGTCGTCTGAGATT |  |  |
| Dlo_030812.1 | *DlPDF1.3*-q-F | TGGCTTCCTCATTCCTGCT | 185 | 60 |
|  | *DlPDF1.3-*q-R | TTGATGGTGTGGACGGTGT |  |  |
| Dlo_011527.1 | *DlBBM*-q-F | AAGCAGCAGAAGAACACGG | 201 | 60 |
|  | *DlBBM-*q-R | TCCCTTGCGAGTTTGTCCT |  |  |
| Dlo_020986.1 | *DlGH3.6*-q-F | CACCACCATCATAACCACAG | 149 | 59 |
|  | *DlGH3.6-*q*-*R | CTGGCACTACGAGAAAGGAT |  |  |
| Dlo_017585.1 | *DlAGL80*-q-F | TCGTCGTCATCATCATTCG | 88 | 59 |
|  | *DlAGL80-*q*-*R | TGTTCCTCATTCCACCAAGA |  |  |
| Dlo_012160.1 | *DlABI3*-q-F | AGCCCAAACCCTAACCACA | 159 | 60 |
|  | *DlABI3-*q-R | TGCCAAAGAGTCACCCGTA |  |  |
| Dlo_020694.1 | *DlPIN1*-q-F | TCTGTGGGAGTTTGATGGTC | 146 | 59 |
|  | *DlPIN1-*q-R | CCTGAATGAAGTGATGGAGC |  |  |
| Dlo_022316.1 | *DlWOX9*-q-F | ACTGTTGCTCCTCCTCCACT | 196 | 60 |
|  | *DlWOX9-*q-R | CTTCACCAAATGCCTCGTG |  |  |
| Dlo_032045.1 | *DlWOX2*-q-F | CTCAAGATGGAACCCGACA | 86 | 60 |
|  | *DlWOX2-*q-R | CCTGCTCTGCTGTTGGTGT |  |  |
| Dlo_026819.1 | *DlGIF2*-q-F | ACCGCAACAACCCTCAATG | 105 | 61 |
|  | *DlGIF2-*q-R | GGGAAAGACGACCTTGGTGT |  |  |
| Dlo_026048.1 | *DlRGF3*-q-F | ACTCTTCTCTGCTGCTTGGA | 176 | 58 |
|  | *DlRGF3-*q-R | TCACCTTTACCTTCTCTGGC |  |  |
| Dlo_013012.1 | *DlLTP*-q-F | TGAGGTCTGGTGGGAGTGTA | 149 | 60 |
|  | *DlLTP-*q*-*R | GGCGAGGTTAGTGTTGATGC |  |  |
| Dlo_004646.1 | *DlPLT2*-q-F | GATTGGAAGAGTGGCAGGA | 176 | 59 |
|  | *DlPLT2-*q*-*R | TTGGAAGGGTGTTGCTTTC |  |  |
| Dlo_019949.1 | *DlLEA5*-q-F | ATGGCTCGTTCTTTCACCA | 108 | 59 |
|  | *DlLEA5-*q-R | TACAGTCCCTTGTGCTCCAG |  |  |
| Dlo_030517.1 | *DlCHI-*q-F | TCTTGCCTTAGCCATTCCC | 170 | 60 |
|  | *DlCHI-*q-R | ACCGAGGATACGCATTGAG |  |  |
